# First-In-Human, Phase 1, Randomized, Dose-Escalation Trial with Recombinant Anti–IL-20 Monoclonal Antibody in Patients with Psoriasis

**DOI:** 10.1371/journal.pone.0134703

**Published:** 2015-08-07

**Authors:** Alice B. Gottlieb, James G. Krueger, Mia Sandberg Lundblad, Marie Göthberg, Brett E. Skolnick

**Affiliations:** 1 Department of Dermatology, Tufts Medical Center, Boston, MA, United States of America; 2 Department of Dermatology, Tufts University School of Medicine, Boston, MA, United States of America; 3 The Rockefeller University, New York, NY, United States of America; 4 Clinical Pharmacology, Novo Nordisk A/S, Søborg, Denmark; 5 Medical-Science, Inflammation, Novo Nordisk Inc., Princeton, NJ, United States of America; Exploratory Oncology Research & Clinical Trial Center, National Cancer Center, JAPAN

## Abstract

**Background:**

The current trial was a first-in-human clinical trial evaluating the safety, tolerability, pharmacokinetics, pharmacodynamics, and preliminary efficacy of the recombinant monoclonal anti−interleukin-20 (IL-20) antibody, NNC0109-0012, which targets the inflammatory cytokine IL-20.

**Methods:**

In total, 48 patients aged 18 to 75 years with moderate to severe stable chronic plaque psoriasis with affected body surface area ≥15% and physician global assessment score ≥3 were enrolled in this randomized, double-blind, multicenter, placebo-controlled, phase 1 dose-escalation trial. Patients were randomized within each single dose cohort (0.01, 0.05, 0.2, 0.6, 1.5, or 3.0 mg/kg) or multiple dose cohort (0.05, 0.2, 0.5, 1.0, or 2.0 mg/kg; 1 dose every other week for 7 weeks) of NNC0109-0012 or placebo in a 3:1 ratio. In the expansion phase, 7 patients were randomized to weekly doses of 2.0 mg/kg NNC0109-0012 or placebo for 7 weeks. The primary objective, safety and tolerability, was assessed by evaluating adverse events (AEs). Additional endpoints included pharmacokinetics, pharmacodynamics, and clinical response (assessed using the Psoriasis Area and Severity Index [PASI] score).

**Results:**

AEs were reported in 85% of patients (n = 40) in the initial study phases (NNC0109-0012, 83%; placebo, 92%) and in 4 of 7 patients in the multiple-dose expansion phase. One serious AE was reported but was judged not to be causally related to NNC0109-0012. No dose-limiting toxicities were reported. NNC0109-0012 pharmacokinetics was similar to other monoclonal antibodies, with an average half-life of approximately 3 weeks. There was a dose-proportional increase in area under the curve and maximum concentration after single dosing. No substantial changes in pharmacodynamic parameters were observed. The expansion phase was terminated early due to apparent lack of PASI improvement.

**Conclusion:**

Single and multiple doses of NNC0109-0012, ranging from 0.05 to 3.0 mg/kg, were well tolerated in patients with psoriasis and exhibited pharmacokinetics similar to that of other monoclonal antibodies.

**Trial Registration:**

ClinicalTrials.gov NCT01261767

## Introduction

Psoriasis (PsO) is an inflammatory disease mediated primarily by T cells and dendritic cells [1]. In PsO, activated T cells migrate to the dermis and release cytokines that result in epidermal hyperplasia, hyperproliferation of keratinocytes, and cutaneous infiltration by immune cells [1, 2]. Plaque PsO, the most common form of PsO, is characterized by scaly, thickened erythematous plaques, usually located on the elbows, knees, scalp, lower back, genitals, palms, and soles of the feet [1, 3]. In the United States, the prevalence of PsO is 1% to 3% [4–7], and the incidence is estimated at 80 per 100,000 person-years [8, 9]. Approximately 17% of all patients with PsO experience moderate to severe disease symptoms (defined as ≥3% affected body surface area [BSA]) [7]. Clinical data have shown that marketed biologic therapies, including adalimumab, etanercept, infliximab, secukinumab [10], and ustekinumab, are associated with a treatment response (ie, achieving 75% improvement from baseline in the Psoriasis Area and Severity Index [PASI] score) in 34% to 88% of patients with moderate to severe PsO [11].

Interleukin (IL)‒20 is a cytokine that appears to be involved in epithelial integrity and host defense [12]. Cultured keratinocytes constitutively express high levels of IL-20 mRNA [13]; furthermore, IL-20 mRNA and protein are upregulated in the keratinocytes of lesional skin from patients with PsO [13–16]. Additional sources of IL-20 include activated monocytes and dendritic cells [17, 18]. IL-20 receptors have not been detected on resting or activated immune cells, including macrophages, monocytes, B cells, T cells, natural killer (NK) cells, and dendritic cells [13, 19]; however, receptors for IL-20 are highly expressed on cultured keratinocytes and in lesional skin from patients with PsO [13, 15, 19]. IL-20 exerts its action through 2 receptor dimers: IL-20R1/IL-20R2 (type I) and IL-22R1/IL-20R2 (type II) [20]. IL-20 promotes hyperproliferation and prevents terminal differentiation of keratinocytes [19, 21, 22].

Overexpression of IL-20 in transgenic mice resulted in a PsO-like skin phenotype, although infiltrating immune cells, seen in human PsO, were not detected [22]. IL-20 belongs to the IL-10 family of cytokines, which also includes IL-19, IL-22, IL-24, and IL-26 [22]; all are involved in host defense [12]. Most IL-20 subfamily members are highly expressed in psoriatic lesions [23]. Neutralizing antibodies to IL-20 significantly reduced epidermal thickness and decreased clinical PsO scores in severe combined immunodeficiency mice transplanted with human PsO xenografts [24].

NNC0109-0012 is a fully human, recombinant immunoglobulin G4 monoclonal antibody that binds to and neutralizes the pharmacologic activity of IL-20. NNC0109-0012 contains an S241P mutation to prevent the formation of half-antibodies. NNC0109-0012 is the first monoclonal antibody targeting IL-20 (anti‒IL-20) and is being considered for the treatment of PsO, rheumatoid arthritis, and other inflammatory diseases. The present study is a first-in-human trial investigating the safety, tolerability, pharmacokinetics (PK), pharmacodynamics (PD), and preliminary efficacy of anti‒IL-20 in patients with PsO.

## Materials and Methods

### Study Design

**Fig 1:**
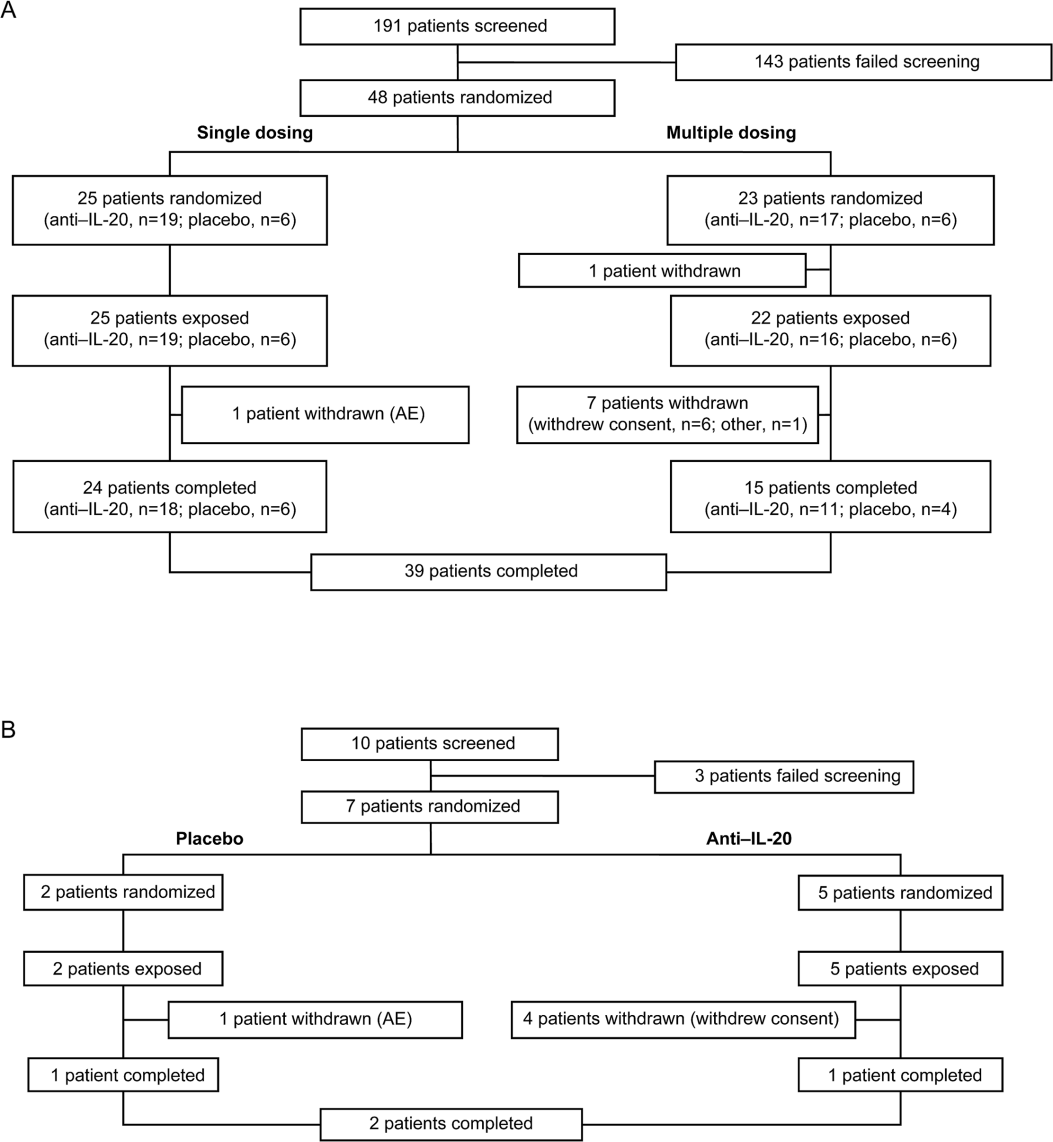
Consort flow diagram. Patient disposition in (A) the single-dose and multiple-dose dose-escalation phases and (B) the multiple-dose expansion phase. Represents the safety analysis set. AE = adverse event.

This was a randomized, double-blind, multicenter, placebo-controlled, phase 1/2a dose-escalation trial (NCT01261767, ClinicalTrials.gov; **Fig 1**; S1 Protocol and S1 CONSORT Checklist) conducted at 13 sites in the United States between April 2008 and August 2010 that investigated the safety and PK (PK data partially published in Lundblad MS, et al 2015[25]) of single and multiple doses of anti‒IL-20. At the time this study was initiated it was a phase 1 study and was thus not required to be registered; however, it was registered once it was decided to initiate the phase 2 (multiple dose) part of the study. The authors confirm that all ongoing and related trials for the anti‒IL20 program are registered. The trial was conducted under an investigational new drug application approved by the US Food and Drug Administration and the central institutional review boards (ie, Baylor Research Institute; Chesapeake Research Review, Inc.; Virginia Clinical Research, Inc.; Western institutional review board [IRB]) and local institutional review boards (ie, New York School of Medicine IRB; Mount Sinai Medical Center for the Protection of Human Subjects; Oregon Health and Science University Research Integrity Office; The University of Utah IRB; Tufts Health Sciences Campus IRB; Wake Forest University Health Sciences IRB), and was performed in accordance with the Declaration of Helsinki and its amendments and the International Conference of Harmonisation Good Clinical Practices [26, 27]. All patients provided written informed consent prior to enrollment into the study.

**Fig 2:**
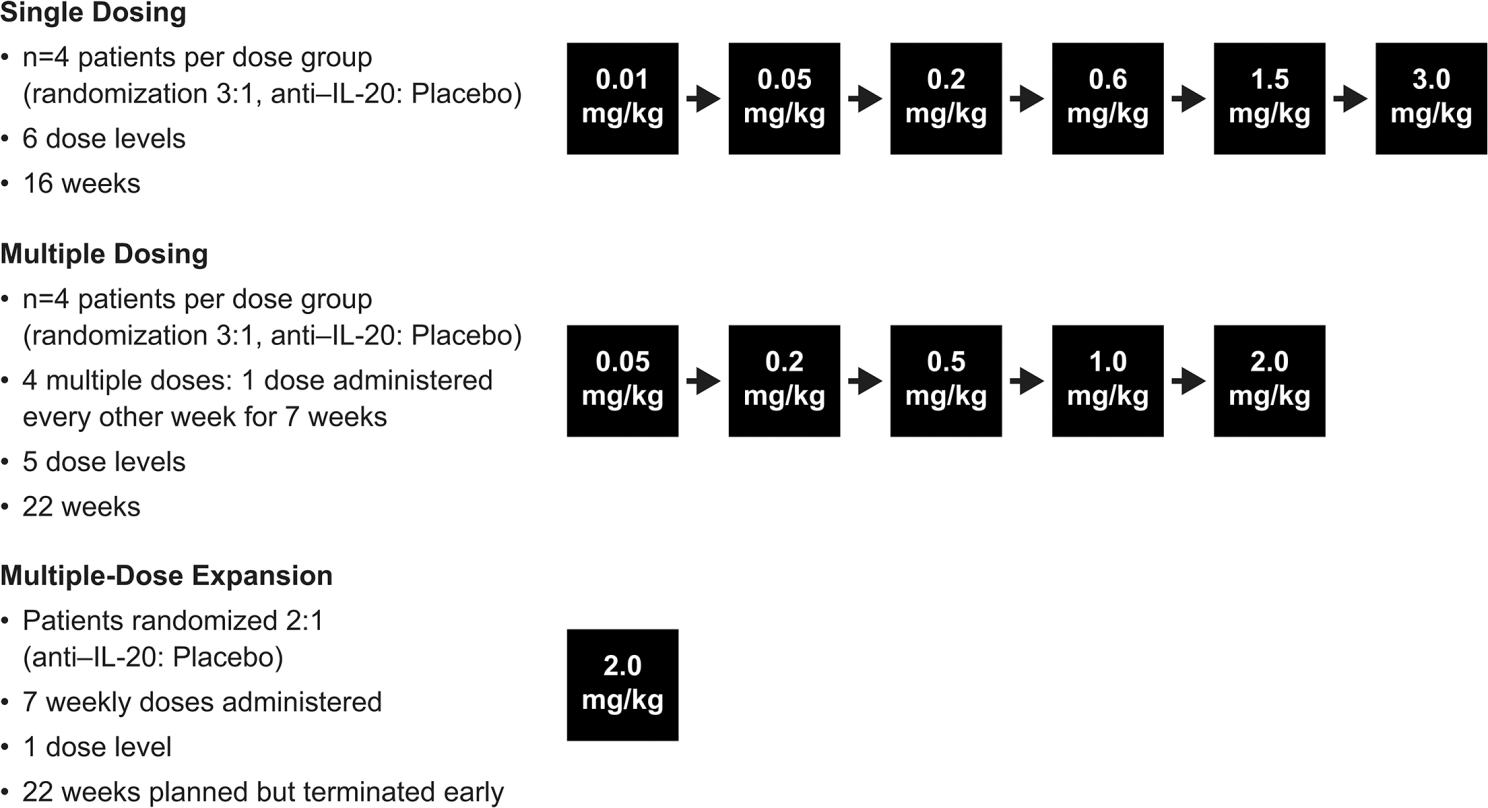
Study design. The first dose in the multiple-dose dose-escalation phase was administered after the third dose level of the single-dose dose-escalation phase (0.2 mg/kg) was evaluated by a study safety group; thereafter, the single- and multiple-dose dose-escalation phases were performed in parallel.

The trial consisted of 3 parts: a single-dose (SD) dose-escalation phase of 16 weeks, a multiple-dose (MD) dose-escalation phase of 22 weeks, and a MD expansion phase of 22 weeks **(Fig 2)**. Patients were assigned numbers manually or via an interactive voice response system and were randomized to anti‒IL-20 or placebo. Study drug was supplied to each trial site in numbered boxes. Each dose cohort, both in the SD and the MD dose-escalation phases, consisted of 4 patients, with 3 receiving active drug and 1 receiving placebo. The trial was double-blinded within each dose cohort, and all relevant staff remained blinded until study completion. The starting dose and dose range were selected based on preclinical studies in cynomolgus monkeys and PK/PD simulations (unpublished studies).

In the SD dose-escalation phase, anti‒IL-20 was administered at 6 different dose levels (0.01, 0.05, 0.2, 0.6, 1.5, and 3.0 mg/kg). In the MD dose-escalation phase, anti‒IL-20 was administered at 5 dose levels (0.05, 0.2, 0.5, 1.0, and 2.0 mg/kg) once every second week for a total of 4 doses. The first dose cohort in the MD dose-escalation phase started after the third dose level (0.2 mg/kg) of the SD dose-escalation phase had been evaluated by the study safety group; thereafter, the SD and MD dose-escalation phases were conducted simultaneously. Patients were assigned to only 1 dose cohort. Safety and PK were evaluated for each dose before initiation of the subsequent dose cohort. The MD expansion phase was only initiated once the PK and safety data from all the SD and MD dose-escalation cohorts had been evaluated. Because no dose-limiting toxicities were observed, the dose selected for the expansion phase was the maximum dose evaluated in the MD dose-escalation phase. Consequently, patients in the MD expansion phase received 7 weekly doses of 2.0 mg/kg anti‒IL-20 or placebo (randomization ratio, 2:1) with a planned cohort of 39 patients. The expansion phase was terminated early because of an apparent lack of effect at the dose and dosing interval studied.

Study drug was administered in the morning as subcutaneous injections in the abdominal wall.

### Patients

Adults aged 18 to 75 years with moderate to severe stable chronic plaque PsO for at least 6 months (with or without psoriatic arthritis), affected BSA ≥15%, physician global assessment (PGA) score ≥3, and who provided written informed consent were included. The main exclusion criteria were forms of PsO other than plaque PsO; concomitant anti-PsO therapy; infectious disease requiring systemic anti-infectious treatment within 2 weeks before study drug administration; known HIV or hepatitis B or C; active herpes, herpes zoster, or cold sores; renal or hepatic insufficiency; lymphoproliferative disease; live virus or bacteria vaccine within the last month before study drug administration; history or signs of malignancy within the last 5 years; childbearing potential in women; or men not using appropriate contraception.

Patients were only allowed to participate in one part of the trial and could not continue from 1 phase to the other. Key inclusion and exclusion criteria for all 3 phases of the trial were identical.

### Safety Assessments

The primary objective was safety and tolerability, which was assessed throughout the duration of the trial by evaluating adverse events (AEs), classified according to the National Cancer Institute Common Terminology Criteria for Adverse Events, version 3, and coded using the Medical Dictionary for Regulatory Activities, version 13.0, including dose-limiting toxicities and local injection-site tolerability. Clinical laboratory parameters, electrocardiogram (ECG), and vital signs were also assessed. In addition, serum antibodies to anti‒IL-20 were measured during the MD dose-escalation phase and the MD expansion phase of the trial.

### Pharmacokinetic Assessments

Pharmacokinetic parameters, including serum concentration, area under the curve (AUC), maximum concentration (C_max_), time to reach C_max_ (t_max_), and terminal half-life (t_½_) of anti‒IL-20, were assessed as secondary endpoints. In the SD dose-escalation phase, serum samples were obtained before dosing on day 1 and at 2, 4, 8, 10, and 24 hours postdose and on day 3 and weeks 2, 3, 5, 9, 13, and 16 postdose. In the MD dose-escalation phase, serum samples were obtained before dosing on days 1, 15, 19, and 43; samples were also obtained at 1, 2, 4, and 24 hours after the first and fourth administration of study drug and on day 2 and weeks 2, 3, 5, 7, 9, 11, 15, 19, and 22. Serum concentrations of anti‒IL-20 were determined using a validated enzyme-linked immunosorbent assay specific for anti‒IL-20. The lower limit of quantification (LLOQ) was 0.2 ng/mL.

### Pharmacodynamic Assessments

As exploratory endpoints, the following PD parameters were assessed: inflammation markers (C-reactive protein [CRP] and erythrocyte sedimentation rate [ESR]), lymphocyte subsets (T cells, CD4^+^ T cells, CD8^+^ T cells, B cells, and NK cells), and a plasma protein biomarker (soluble CD25). Lesional biopsies were optional and obtained from MD patients (dose-escalation phase and expansion phase) before dosing and at weeks 3 and 15; nonlesional biopsies were obtained only at baseline. Patient biopsies were evaluated in a blinded fashion. Clinical effect was the primary objective in the MD expansion phase and was evaluated using the PASI (total score, 0−72) [28, 29]; improvement was defined as a 75% change in PASI from baseline (PASI75). In the SD and MD dose-escalation phases, assessment of clinical effect was exploratory only.

### Statistical Analysis

The full analysis set, which included all randomized patients receiving treatment, was used for the analysis and data presentations. The primary endpoint (AEs) was presented using descriptive statistics. The number of AEs and the number and percentage of patients who experienced ≥1 AE were summarized.

Pharmacokinetic parameters for anti‒IL-20 were derived by using noncompartmental methods. AUC was calculated using the linear trapezoidal method on measured concentrations and actual time points. AUC was calculated as the sum of the area under the concentration-time curve from zero to the last valid concentration (AUC_0−t_) and the area from last valid concentration to infinity (AUC_t−∞_). AUC_t−∞_ was approximated by C_t_/λ_z_, where C_t_ was the estimated last concentration and λ_z_ was the terminal rate constant. The t_½_ was estimated as ln(2)/λ_z_. The accumulation index (R_acc_) was calculated as the AUC_0−336h_ after administration of the fourth dose divided by the AUC_0−336h_ after administration of the first dose of the trial product in the MD dose-escalation phase. For the MD expansion phase, R_acc_ was calculated as the AUC_0−168h_ after administration of the seventh dose divided by the AUC_0−168h_ after administration of the first dose. Dose proportionality was investigated by fitting a linear regression model to the log-transformed endpoint with log-dose as continuous covariate.

The PASI score obtained 2 weeks after dosing in the SD phase and 8 weeks after the initial dose in the MD phases was investigated by applying an analysis of variance model with dose as fixed factor and the baseline level as continuous covariate.

The number of patients in each dose cohort was based on consideration of safety assessments; no formal sample size calculations were performed.

## Results

### Patient Disposition and Baseline Characteristics

In the SD and MD dose-escalation phases, 48 patients were randomized, 47 were dosed, and 39 completed the trial **(Fig 1A)**. In the SD dose-escalation phase, 1 patient withdrew from the study because of AEs. In the MD-dose escalation phase, 1 patient was withdrawn before being given treatment, and 7 patients withdrew consent after being given treatment. In the SD dose-escalation phase, 4 patients received 0.05 mg/kg anti‒IL-20 (1 patient withdrew because of deteriorating PsO but was replaced), and only 2 patients received 1.5 mg/kg anti‒IL-20 (1 patient randomized to 1.5 mg/kg anti‒IL-20 received a 3.0-mg/kg dose in error). In the MD expansion phase, 39 patients were planned to be included (26 patients allocated to anti‒IL-20 and 13 patients to placebo). However, the MD expansion phase was terminated early because of a lack of apparent effect at the tested dose; consequently, only 10 patients were screened for the MD expansion phase, 7 patients were given treatment, and only 2 patients completed the trial **(Fig 1B)**.

Baseline demographics and clinical disease characteristics are shown for the SD dose-escalation phase in **Table 1**and for the MD dose-escalation phase and the MD expansion phase in **Table 2**.

**Table 1:** Patient and Disease Characteristics for Patients Receiving Single Doses.

		Anti‒IL-20, mg/kg					
	Placebo (n = 6)	0.01 (n = 3)	0.05 (n = 4)	0.20 (n = 3)	0.60 (n = 3)	1.5 (n = 2)	3.0 (n = 4)
Mean (range) age, y	41 (24–57)	51 (46–58)	46 (29–63)	51 (44–59)	27 (22–33)	21 (20–21)	52 (47–55)
Female/male, n	1/5	0/3	2/2	1/2	0/3	0/2	2/2
Race, n (%)							
White	4 (67)	3 (100)	4 (100)	2 (67)	3 (100)	2 (100)	4 (100)
Mean (range) weight, kg	87 (52–109)	102 (92–115)	84 (66–93)	93 (83–99)	87 (70–100)	82 (63–101)	78 (59–97)
Mean (range) BSA affected, %	27 (15–50)	38 (25–50)	19 (17–22)	30 (15–42)	28 (15–40)	29 (15–43)	25 (15–40)
Mean (range) PASI total score	19 (13–28)	27 (12–42)	18 (10–25)	18 (8–34)	33 (26–38)	22 (6–37)	23 (6–42)
Mean (range) duration of psoriasis, y	23 (1–56)	12 (4–23)	17 (4–33)	22 (17–29)	11 (4–18)	10 (9–11)	28 (4–39)
Psoriatic arthritis, n (%)	1 (17)	0 (0)	0 (0)	0 (0)	0 (0)	0 (0)	0 (0)

BSA = body surface area; IL-20 = interleukin 20; PASI = Psoriasis Area and Severity Index score.

**Table 2:** Patient and Disease Characteristics for Patients Receiving Multiple Doses.

	Dose-Escalation Phase						Expansion Phase	
		Anti‒IL-20, mg/kg						
	Placebo (n = 6)	0.05 (n = 3)	0.20 (n = 3)	0.50 (n = 4)	1.0 (n = 3)	2.0 (n = 3)	Placebo (n = 2)	Anti‒IL-20 2.0 mg/kg (n = 5)
Mean (range) age, y	34 (19–60)	40 (22–57)	37 (30–40)	37 (27–51)	32 (23–51)	43 (30–52)	46 (33–58)	44 (28–60)
Female/male, n	0/6	1/2	0/3	0/4	0/3	0/3	1/1	0/5
Race, n								
White	6 (100)	3 (100)	3 (100)	3 (75)	2 (67)	3 (100)	1 (50)	5 (100)
Mean (range) weight, kg	97 (78–123)	105 (95–115)	88 (75–97)	83 (75–88)	102 (91–116)	107 (93–122)	73 (59–87)	94 (72–104)
Mean (range) BSA affected, %	33 (18–70)	31 (20–48)	20 (15–29)	47 (23–81)	36 (32–40)	17 (15–21)	28 (26–29)	33 (17–60)
Mean (range) PASI total score	18 (13–27)	24 (17–37)	15 (14–17)	29 (16–36)	19 (17–21)	15 (11–19)	16 (12–20)	19 (15–24)
Mean (range) duration of psoriasis, y	16 (3–26)	29 (19–48)	17 (5–24)	9 (4–12)	22 (7–47)	25 (20–30)	22 (19–26)	12 (1–30)
Psoriatic arthritis, n (%)	3 (50)	1 (33)	1 (33)	3 (75)	0 (0)	0 (0)	2 (100)	3 (60)

BSA = body surface area; IL-20 = interleukin 20; PASI = Psoriasis Area and Severity Index score.

### Safety and Tolerability

During the SD and MD dose-escalation phases, AEs were reported in 85% of patients (anti‒IL-20, 83%; placebo, 92%; **Table 3**). The most common AEs reported with anti‒IL-20 during the SD dose-escalation phase were increased ESR (32% of patients), headache (16%), and pruritus (16%). The most common AEs reported with anti‒IL-20 in the MD dose-escalation phase were increased ESR (25% of patients), increased uric acid (19%), and oropharyngeal pain (19%). Overall, in the SD and MD dose-escalation phases, the majority (133/181; 73%) of AEs were of mild severity. Seven AEs were rated as severe (SD dose-escalation phase, n = 2; MD dose-escalation phase, n = 5); all were considered unlikely to be related to treatment by the investigator except 1 non–life-threatening AE (hyperuricemia) in a patient receiving 2.0 mg/kg anti‒IL-20 in the MD dose-escalation phase. One event in the MD dose-escalation phase was considered life threatening (hyperuricemia; placebo group). One serious AE (SAE) was reported in the trial, during the MD dose-escalation phase (moderate cellulitis reported 19 days after the fourth and final dose of 0.05 mg/kg anti‒IL-20), but this SAE was judged as having no causal relationship to the study drug. Only 1 AE (increase in psoriatic plaques) led to withdrawal from the trial (SD dose-escalation phase, 0.05 mg/kg anti‒IL-20 group).

**Table 3:** Treatment-Emergent AEs by Organ Class Occurring in >1 Patient in Any Study Group.

	Single-Dose Dose-Escalation Phase				Multiple-Dose Dose-Escalation Phase				Multiple-Dose Expansion Phase			
	Placebo(n = 6)		Anti‒IL-20 (n = 19)		Placebo(n = 6)		Anti‒IL-20 (n = 16)		Placebo (n = 2)		Anti‒IL-20 (n = 5)	
	n (%)	E	n (%)	E	n (%)	E	n (%)	E	n (%)	E	n (%)	E
AEs	5 (83)	17	15 (79)	73	6 (100)	21	14 (88)	70	2 (100)	10	2 (40)	7
Investigations	1 (17)	3	8 (42)	12	3 (50)	10	8 (50)	16	0	0	1 (20)	1
Gastrointestinal disorders	1 (17)	3	6 (32)	8	1 (17)	1	3 (19)	3	1 (50)	1	0	0
Nervous system disorders	1 (17)	1	5 (26)	8	2 (33)	2	4 (25)	6	0	0	1 (20)	1
Skin and subcutaneous tissue disorders	2 (33)	4	5 (26)	8	0	0	1 (6)	1	1 (50)	1	2 (40)	5
Musculoskeletal and connective tissue disorders	0	0	5 (26)	7	0	0	3 (19)	4	1 (50)	1	0	0
Infections and infestations	1 (17)	1	5 (26)	6	3 (50)	3	4 (25)	6	1 (50)	1	0	0
Metabolism and nutrition disorders	0	0	5 (26)	6	0	0	6 (38)	11	0	0	0	0
Respiratory, thoracic, and mediastinal disorders	1 (17)	1	4 (21)	5	2 (33)	2	5 (31)	7	2 (100)	3	0	0
General disorders and administration site conditions	1 (17)	2	3 (16)	4	1 (17)	1	3 (19)	3	1 (50)	3	0	0
Injury, poisoning, and procedural complications	2 (33)	2	3 (16)	3	2 (33)	2	3 (19)	6	0	0	0	0
Vascular disorders	0	0	2 (11)	3	0	0	0	0	0	0	0	0

AEs = adverse events; E = number of adverse events reported; IL-20 = interleukin 20.

Safety data from the MD expansion phase were similar to those in the MD dose-escalation phase **(Table 3)**. A total of 17 AEs were reported in 4 patients (placebo, 10 AEs in 2 patients; anti‒IL-20, 7 AEs in 2 patients). All of the events in the MD expansion phase were of mild (6/17; 35%) or moderate severity (11/17; 65%), and no SAEs were reported. Only 1 AE (worsening of psoriasis in a placebo-treated patient) led to study withdrawal. No dose-limiting toxicities were reported in either of the dose-escalation phases or the expansion phase. Therefore, a maximum tolerated dose could not be determined in this trial. No clinically relevant changes were observed in clinical laboratory parameters, ECG, or vital signs in the dose-escalation phases or the expansion phase. In addition, no antibodies to anti‒IL-20 were detected.

### Pharmacokinetics

#### Single dose

**Fig 3:**
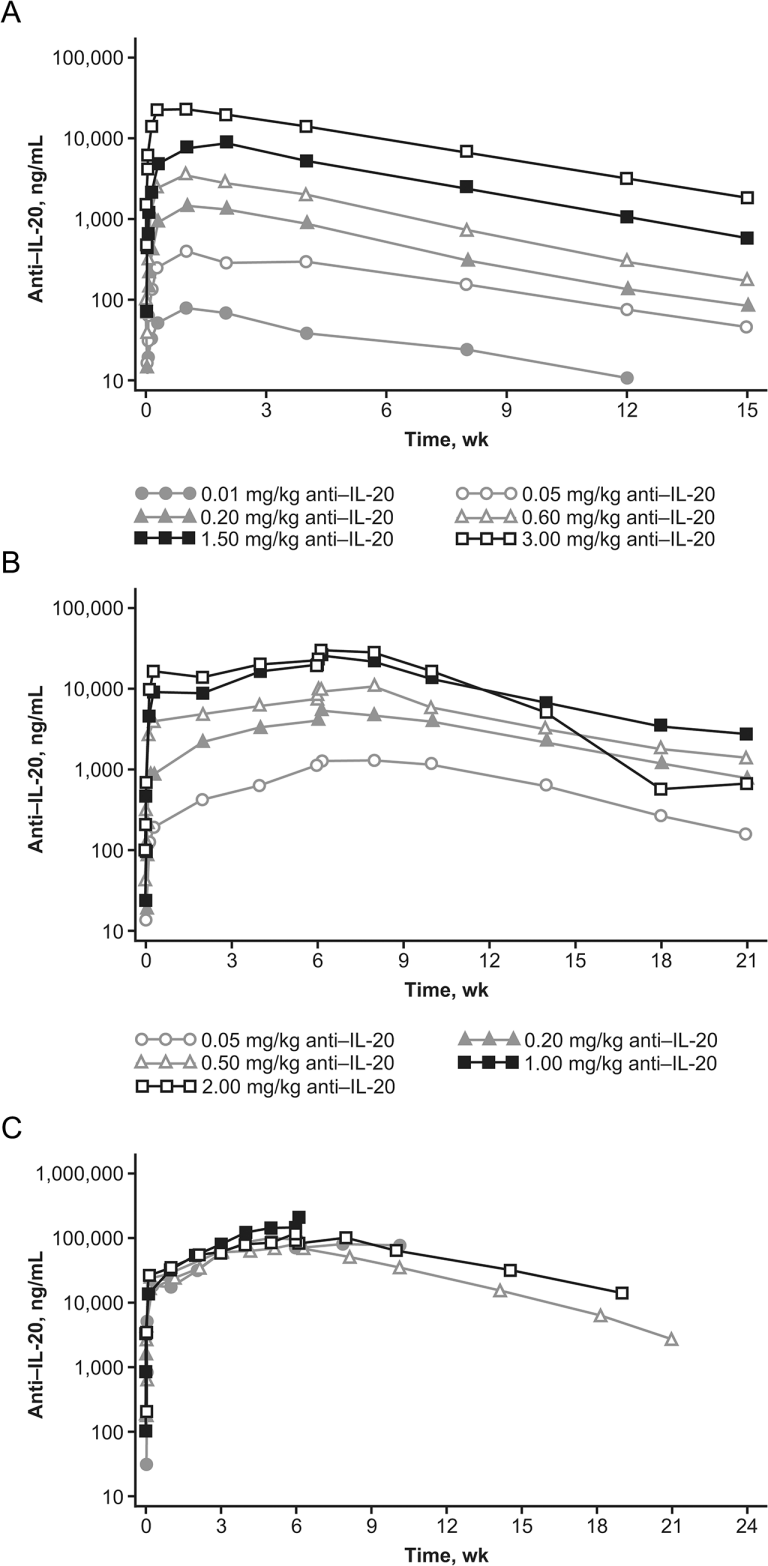
Mean anti‒IL-20 serum concentration-time profiles. Mean serum concentration-time profiles following (A) single dosing or (B) multiple dosing in the dose-escalation phase and individual anti‒IL-20 serum concentration-time profiles following multiple dosing in (C) the expansion phase on a semi-logarithmic scale in patients with psoriasis.

A dose-proportional increase in anti‒IL-20 AUC (range, 82.1−25,143 μg∙h/mL) and C_max_ (range, 0.08−24.09 μg/mL) was observed in the tested dose range (**Fig 3A** and **Table 4**). Serum concentrations of anti‒IL-20 were above LLOQ (0.2 ng/mL) for a mean of 82 days for the 0.05-mg/kg dose and for a mean of 104 to 108 days for the other doses. The mean t_max_ ranged from 4 to 15 days across the dose levels, and the mean t_½_ ranged from 21 to 27 days.

**Table 4:** Summary of Pharmacokinetic Parameters After Dosing With Anti‒IL-20.

		Geometric Mean (CV%)					
		C_max_, μg/mL		AUC, μg∙h/mL		t_½_, h	
Anti‒IL-20 Dose, mg/kg	Dose Number	n	Value	n	Value	n	Value
Single dose							
0.01	1	3	0.08 (53)	3	82 (40)	3	631 (12)
0.05	1	4	0.41 (27)	3	500 (31)	3	641 (14)
0.2	1	3	1.43 (36)	3	1496 (34)	3	536 (7)
0.6	1	3	3.37 (49)	3	3311 (37)	3	507 (7)
1.5	1	2	8.79 (5)	2	9203 (5)	2	580 (2)
3.0	1	4	24.09 (38)	4	25,143 (30)	4	616 (13)
Multiple doses							
0.05	4	3	1.08 (44)	2	1848 (21)	2	629 (3)
0.2	4	3	3.88 (26)	2	6720 (7)	2	772 (4)
0.5	4	3	9.19 (28)	2	10,900 (39)	2	594 (30)
1.0	4	3	19.96 (32)	3	22,712 (30)	3	767 (16)
2.0	4	3	23.62 (48)	3	20,373 (60)	3	353 (32)

AUC = area under the curve; C_max_ = maximum concentration; CV = coefficient of variation; IL-20 = interleukin 20; t_½_ = half-life.

#### Multiple dose

Depending on dose, R_acc_ for anti‒IL-20 ranged from 2.0 to 4.1, and steady state was not achieved after the fourth and final dose **(Fig 3B and Table 4)**. The mean terminal t_1/2_ ranged from 15 to 32 days. The PK results from the 5 patients in the MD expansion phase who received active trial drug suggest that a steady state had not been reached after the 7 weekly doses of 2 mg/kg of anti‒IL-20 **(Fig 3C)**. The terminal t_1/2_ of anti‒IL-20 was 21 and 30 days, respectively, in the 2 patients for whom data were available. Following dose 7, geometric mean C_max_ was 8.89 μg/mL and geometric mean AUC was 7126 μg∙h/mL.

### Pharmacodynamics

No significant or clinically relevant changes in ESR, CRP, lymphocyte subsets, or soluble CD25 were observed after dosing with anti‒IL-20 in the SD or MD dose-escalation phases or the MD expansion phase of the trial. Biopsy samples were obtained from 13 of 22 (59%; anti‒IL-20, n = 10; placebo, n = 3) patients in the MD dose-escalation phase and 3 patients (anti‒IL-20, n = 1; placebo, n = 2) in the MD expansion phase. Because of the limited number of biopsy samples, no definitive conclusions regarding this exploratory endpoint can be formed.

### Efficacy

**Fig 4:**
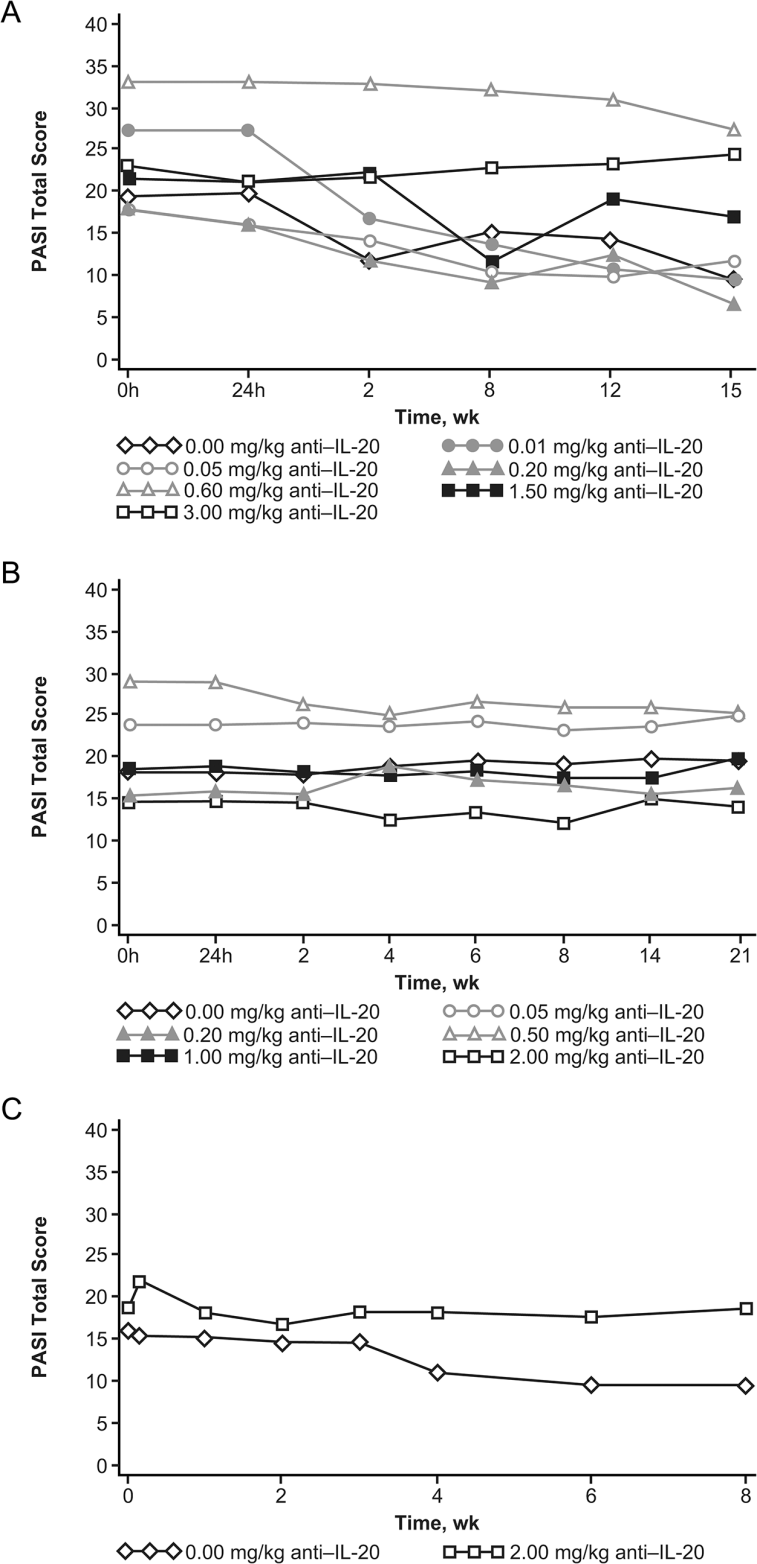
Mean PASI total score by visit. Mean PASI total score by visit following (A) single dosing or (B) multiple dosing in the dose-escalation phase and (C) multiple dosing in the expansion phase. PASI = Psoriasis Area and Severity Index.

No significant dose-dependent changes in total PASI score were observed with single or multiple doses of anti‒IL-20 compared with placebo in the dose-escalation phases or the MD expansion phase **(Fig 4)**. Following single doses, 1 of 6 patients receiving placebo and 4 of 19 patients receiving anti‒IL-20 (1.5 or 3.0 mg/kg) achieved a PASI75 score. Following multiple doses in the dose-escalation phase, none of the 6 patients receiving placebo and only 1 of the 16 patients receiving anti‒IL-20 (0.5 mg/kg) achieved a PASI75 score. In the MD expansion phase, none of the 7 patients achieved a PASI75 score at any visit.

## Discussion

In this first-in-human trial, the safety, tolerability, PK, PD, and preliminary clinical response of the fully human anti−IL-20 monoclonal antibody NNC0109-0012 were evaluated in patients with PsO. Single doses (0.01−3.0 mg/kg) and multiple doses (0.05−2.0 mg/kg) of anti‒IL-20 were well tolerated, with no clinically relevant changes in clinical laboratory values, ECG, physical examination, or vital signs, and no AEs were attributed to anti‒IL-20. No antibodies against anti‒IL-20 were detected. Only 1 SAE was reported (in the MD dose-escalation phase), but it was considered unlikely to be related to the study drug. There were no dose-limiting toxicities, and thus, no maximum tolerated dose could be determined. Because of the subcutaneous route of administration, doses higher than 3.0 mg/kg (which would have required larger injection volumes or additional injections) were avoided. Dose-proportional PK was observed after single doses. No clinically relevant changes in any inflammation biomarker were observed with anti‒IL-20 compared with placebo. Furthermore, no evidence of clinical response was observed in patients with PsO after 7 weekly doses of 2 mg/kg anti‒IL-20.

**Fig 5:**
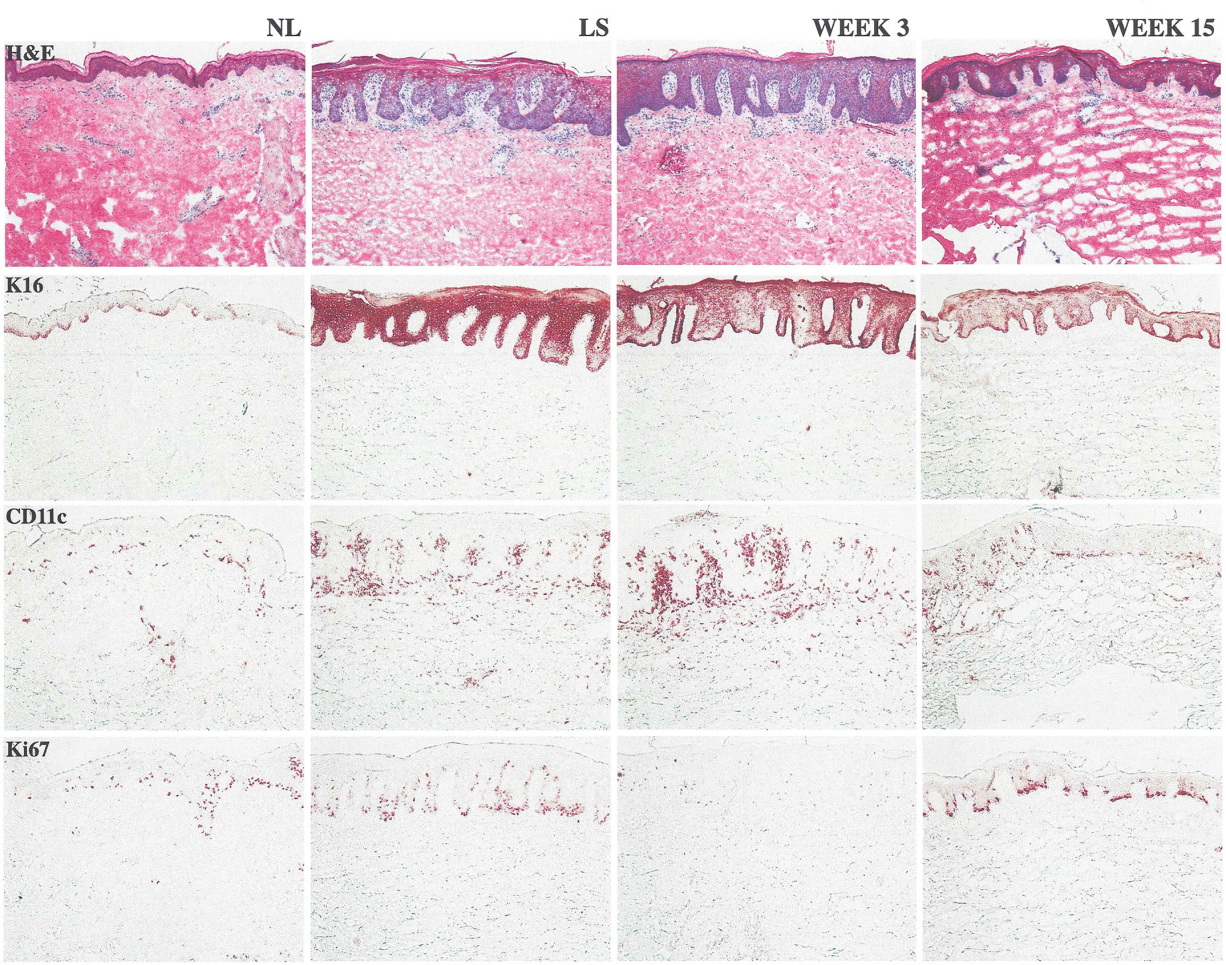
Histology image from a single patient. Patient from the expansion phase treated with anti‒IL-20. By week 15, this patient experienced marked improvement in epidermal hyperplasia and acanthosis, a large reduction in keratin 16, associated reductions in proliferating keratinocytes (Ki67), and marked reductions in CD11c+ dendritic cells. CD11c = integrin alpha X chain protein; H&E = hematoxylin and eosin stain; K16 = keratin 16; Ki67 = cellular marker for proliferation; NL ***=*** nonlesional (skin); LS = longitudinal section.

Histology measurements and evaluation of the responses were performed in a blinded manner. Findings from a single patient in the expansion phase treated with anti‒IL-20 showed marked improvement in epidermal hyperplasia and acanthosis by week 15 **(Fig 5)**. However, the histology analyses could not be used to provide definitive conclusions on exploratory PD endpoints because of the limited number of biopsy samples.

The apparent lack of clinical response in patients with PsO in the dose range of anti‒IL-20 studied could potentially be related to suboptimal doses and/or dosing duration, and it should be noted that the dose-escalation phases were not primarily designed for efficacy. Also, functional redundancies of IL-20 subfamily cytokines in PsO may have contributed to the lack of clinical signs [30]. Studies performed in keratinocytes showed that IL-19, IL-20, IL-22, and IL-24 each induced hyperplasia and epidermal thickening independent of other IL-20 subfamily members. In addition, these cytokines produced similar gene expression signatures, including similar induction or repression of PsO-associated genes as IL-20 [19]. Similar to the current trial, a previous clinical study that targeted IL-22 in patients with PsO (NCT01010542, ClinicalTrials.gov) was terminated early because of a determination that the primary efficacy endpoints (change in PASI, target lesion, and PGA scores) were unlikely to be met. These data support the hypothesis for a role of the IL-20 subfamily on hyperplasia in PsO but suggest that more than 1 subfamily member may need to be inhibited to show a relevant clinical response in patients with PsO. Although a preclinical study in mice transplanted with human psoriatic plaques or nonlesional psoriatic skin plus activated peripheral blood mononuclear cells showed improvement in the semiquantitative clinical psoriasis scores following treatment with an anti‒IL-20 antibody, suggesting that targeting IL-20 may improve PsO symptoms in humans [24], this response is not necessarily predictive of efficacy in humans with PsO because there is no animal model specific to PsO [31]. The importance of IL-20 in psoriasis is further supported by the observation that a specific single nucleotide polymorphism in the promoter region of the IL-20 gene was associated with psoriasis progression [32].

This was primarily a trial designed to investigate the safety and tolerability of anti‒IL-20 and, hence, several limitations of the current trial should be noted, which precluded definitive conclusions regarding clinical response. Most important were the small patient sample size (the cohort sizes were limited because the aim of the dose-escalating phases was not to demonstrate clinical efficacy), limited data (particularly for the histology analyses), and the premature discontinuation of the trial. In addition, a maximum tolerated dose was not reached, suggesting that an optimal dose and dose regimen were not achieved in this trial.

In conclusion, single and multiple doses of the anti‒IL-20 monoclonal antibody NNC0109-0012 were well tolerated in patients with PsO in this first-in-human dose-escalation trial. No dose-limiting toxicities were observed, and no antibodies to anti‒IL-20 were detected. PK properties were similar to other monoclonal antibodies directed against soluble targets. Because of an apparent lack of improvement in PASI scores at the doses studied, the expansion phase was terminated early. NNC0109-0012 is no longer under clinical development.

## Supporting Information

S1 CONSORT Checklist CONSORT 2010 Checklist. (DOC)

S1 Protocol Redacted Study Protocol. (PDF)
