# Supplementary material for: First-In-Human, Phase 1, Randomized, Dose-Escalation Trial with Recombinant Anti–IL-20 Monoclonal Antibody in Patients with Psoriasis
Source: PLoS One. 2015 Aug 7;10(8):e0134703. doi: 10.1371/journal.pone.0134703 (PMC4529098; doi:10.1371/journal.pone.0134703)
Supplement: S1 Protocol — (PDF) [file pone.0134703.s002.pdf]

Protocol

Trial ID: NN8226-1848

A randomised, double-blind, placebo-controlled, single- and multiple dose, dose-escalation trial of Anti-IL-20 (109-0012) 100 mg/vial in psoriatic subjects, followed by an expansion phase.

Trial phase: 1/2a

Author:

[redacted]

Clinical Pharmacology

This confidential document is the property of Novo Nordisk. No unpublished information contained herein may be disclosed without prior written approval from Novo Nordisk. Access to this document must be restricted to relevant parties.

Table of contents

Page

Table of contents 2

Table of figures 6

Table of tables 6

List of abbreviations 7

1 Summary 10

2 Flow charts 14

3 Introduction 18
3.1 Basic information 18
3.1.1 Psoriasis 18
3.1.2 Existing treatment 18
3.1.3 IL-20 19
3.1.4 Trial drug 20
3.2 Rationale for the trial 20

4 Objectives and endpoints 21
4.1 Objectives 21
4.2 Endpoints 22

5 Trial design 25
5.1 Type of trial 25
5.2 Rationale for trial design 26
5.3 Treatment of subjects 27
5.4 Rationale for treatment 29

6 Trial population 31
6.1 Number of subjects to be studied 31
6.2 Inclusion criteria 31
6.3 Exclusion criteria 31
6.4 Withdrawal criteria 33
6.5 Subject replacement 33
6.6 Rationale for trial population 33

7 Trial schedule 35

8 Methods and assessments 36
8.1 Visit procedures 36
8.1.1 Visit procedures for SD dose-escalation phase 36
8.1.2 Visit procedures for MD dose-escalation phase 39
8.1.3 Visit procedures for MD expansion phase 44
8.1.4 Recordings 49
8.1.5 Subject related information and assessments 49
8.2 Assessments for safety and tolerability 50

8.2.1 Adverse events 50
8.2.2 Vital signs 51
8.2.3 12-lead ECG 51
8.2.4 Laboratory safety parameters 51
8.2.4.1 Handling of blood and urine samples 52
8.2.5 Immunogenicity 53
8.2.5.1 Handling of immunogenicity samples 53
8.2.6 Physical examination 53
8.3 Assessments for efficacy 54
8.3.1 Body surface area (BSA) 54
8.3.2 Psoriasis Area and Severity Index (PASI) 54
8.3.3 Physicians Global Assessment (PGA) score 55
8.3.4 Visual Analogue Scale (VAS) 56
8.3.5 Quality of life, DLQI 56
8.4 Other assessments 56
8.4.1 Pharmacokinetic samples 58
8.4.1.1 Sampling and handling (PK samples) 58
8.4.1.2 Blood Sampling Procedure and Sample Handling (PK samples) 58
8.4.1.3 Serum Sample Labelling (PK samples) 59
8.4.1.4 Shipment of Samples (PK samples) 59
8.4.2 Pharmacodynamic Biomarker Assessments 59
8.4.3 Pharmacogenomic Biomarker Assessments 60
8.4.4 Reporting of pharmacodynamic and pharmacogenomic biomarker assessments 61
8.4.5 Sampling and handling of blood, serum, plasma and tissue samples 61
8.4.5.1 Serum samples for biomarker analyses 61
8.4.5.2 Labelling of Serum Samples 61
8.4.5.3 Plasma Samples for Biomarker Analyses 61
8.4.5.4 Blood Samples for Gene Expression Analyses 62
8.4.5.5 Blood Samples for Genotyping 62
8.4.5.6 Tissue Samples for Biomarker Analyses 62
8.4.6 PK/PD modelling 63
8.5 Subject compliance 63

9 Trial supplies 64
9.1 Trial products 64
9.2 Packaging and labelling of trial products 64
9.3 Storage and drug accountability of trial products 65
9.4 Auxiliary supply 65

10 Randomisation, breaking of blinded codes and interactive voice response system (IVRS) 66
10.1 Randomisation and blinding 66
10.2 Breaking of blinded codes 66
10.3 Interactive voice response system (IVRS) 67

11 Concomitant illnesses and medication 68

12 Adverse events 69
12.1 Definitions 69
12.1.1 Defintion of dose limiting toxicities 71

12.1.2 Definition of maximum tolerated dose and maximum administered dose 71
12.2 Collection, recording and reporting of adverse events 72
12.2.1 Medical events of special interest 73
12.3 Follow-up of adverse events 73
12.4 Pregnancy 74
12.5 Precautions/over-dosage 74
12.6 Safety committees 74
12.6.1 Internal Novo Nordisk safety committee 74

13 Case report forms 76
13.1 Rules for completing eCRFs 76
13.2 Corrections to eCRFs 76
13.3 eCRF flow 76

14 Monitoring procedures 77

15 Data management 78

16 Evaluability of subjects for analysis 79

17 Statistical considerations 80
17.1 Sample size calculation 80
17.2 Statistical methods 81
17.2.1 Primary endpoint 81
17.2.2 Secondary endpoints 81
17.3 Interim analysis 82
17.4 Explorative statistical analysis for pharmacogenetics and biomarkers 83
17.5 PK and/or PD modelling 83

18 Ethics 84
18.1 Informed consent form for trial subjects 84
18.2 Data handling 85
18.3 Institutional review boards/independent ethics committee 85
18.4 Regulatory authorities 86

19 Premature termination of the trial 87

20 Protocol compliance 88

21 Critical documents 89

22 Responsibilities 90

23 Reports and publications 91
23.1 Communication and publication 91
23.1.1 Authorship 91
23.1.2 Publication(s) 92
23.1.3 Site-specific publication(s) by Investigator(s) 92
23.2 Investigator access to data and review of results 93

24 Retention of clinical trial documentation 94

25 Indemnity statement 95

26 References 96

**Appendix A**	**Approval of final protocol**
**Appendix B**	**Agreement on the protocol**
**Attachment I - global**	**List of key staff and relevant departments**
**Attachment II**	**Common Terminology Criteria for Adverse Events, v 3.0 (CTCAE)**

Table of figures

Page

Figure 5–1 : Illustration of the concept of the FHD trial, showing the SD phase at six dose levels, the MD phase at five dose levels, and an MD expansion phase at one dose level. 26

Table of tables

Page

Table 2–1 Flow chart for single dose, dose-escalation phase 14
Table 2–2 Flow chart for multiple dose, dose-escalation, and expansion phases 16
Table 5–1 Dose levels for the SD and MD dose-escalation phases and 28
Table 5–2 Dose-escalation rules 30
Table 8–1 PGA score definition 55
Table 8–2 Samples and assays 57
Table 12–1 Severity and toxicity grade of events not covered by CTCAE 71
Table 17–1 Sample size calculation for the MD expansion phase 80

List of abbreviations

15D2-S241P	The fully human anti-IL-20 monoclonal antibody: 1400-250-15D2.B7.G10 with a single point mutation (Ser241Pro) introduced to reduce the formation of half antibodies
AE	Adverse events
ALT	Alanine aminotransferase
ALP	Alkaline phosphatase
AST	Aspartate aminotransferase
AUC	Area under the serum concentration versus time curve from time 0 to infinity
AUC_{0-t}	Area under the serum concentration curve from time 0 to time t
$AUC_{0-\tau}$	Area under the serum concentration curve in the dosing interval
BMI	Body mass index
BP	Blood pressure
BSA	Body surface area
CL	Clearance
C_{max}	Maximum observed serum concentration
COX-2	Cyclooxygenase inhibitor-2
CRO	Contract research organisation
CRP	C-reactive protein
CTCAE	Common terminology criteria for adverse events
DMARD	Disease modifying anti-rheumatic drug
DLT	Drug limiting toxicity
DLQI	Dermatology life quality index
DNA	Deoxynucleotide acid
DUN	Dispensing unit number
EC_{50}	Half maximal effective concentration
eCRF	Electronic case report form
ECG	Electrocardiogram
EDC	Electronic data capture
ESR	Erythrocyte sedimentation rate
FDA	Food and Drug Administration
FHD	First human dose
FSH	Follicle stimulating hormone
GCP	Good clinical practice
HDL	High density lipoprotein
HIV	Human immunodeficiency virus

HR	Heart rate
IB	Investigator's Brochure
ICH	International Conference on Harmonisation
IEC	Independent ethics committee
IFN	Interferon
IgG	Immunoglobulin G
IHC	Immunohistochemistry
IL	Interleukin
IRB	Institutional review board
i.v.	Intravenous
IVRS	Interactive voice response system
K_d	Dissociation constant
LAR	Legally authorised representative
LDL	Low density lipoprotein
M	Molar
MABEL	Minimal anticipated biological effect level
mAb	Monoclonal antibody
MAP	Modelling Analysis Plan
MedDRA	Medical Dictionary for Regulatory Activities
MD	Multiple doses
mRNA	Messenger ribonucleic acid
MTD	Maximum tolerated dose
NCA	Non-compartmental analysis
NCI	National Cancer Institute
NNAS	Novo Nordisk A/S
NNI	Novo Nordisk Incorporated
PASI	Psoriasis Area and Severity Index
PASI75	Improvement in Psoriasis Area and Severity Index score by 75%
PCR	Polymerase chain reaction
PD	Pharmacodynamics
PG	Pharmacogenomics
PGA	Physician's Global Assessment Score
PK	Pharmacokinetics
PUVA	Psoralen+ultraviolet light A
pSTAT3	Phosphorylated signal transducer and activator of transcription 3
QA	Quality assurance

qRT-PCR	Quantifiable reverse transcription polymerase chain reaction
RA	Rheumatoid arthritis
R_{acc}	Accumulation index
s.c.	Subcutaneous
SAE	Serious adverse event
SD	Single dose
SNP	Single nucleotide polymorphism
$t_{1/2}$	Terminal serum half-life
t_{max}	Time to maximum observed serum concentration
TMM	Trial Materials Manual
TNF	Tumour necrosis factor
TSH	Thyroid stimulating hormone
ULN	Upper limits of normal
UV	Ultra violet
VAS	Visual analogue scale

1 Summary

Anti-interleukin-20 monoclonal antibody 15D2-S241P (mAb), is a fully human, recombinant monoclonal antibody of immunoglobulin G_4 (IgG_4) isotype, containing one point mutation (S241P). The actual drug substance is named NNC 0109-0000-0012 (hereinafter abbreviated 109-0012) and the drug product is named Anti-IL-20 (109-0012) 100 mg/vial. In this First Human Dose (FHD) trial, subcutaneous doses of the drug product will be administered to psoriatic subjects for the first time, in order to establish a safe dose range for subsequent clinical trials.

The FHD trial will be a double-blind, placebo-controlled phase 1/2a investigation with a single dose (SD) dose-escalation phase and a multiple dose (MD) dose-escalation phase, in semi-parallel. The aim is to investigate 6 single and 5 multiple dose levels in the SD and MD dose-escalation phases, respectively. In the SD dose-escalation phase subjects will receive their one and only dose at Day 1 and will thereafter be followed through trial Week 16. During the MD dose-escalation phase subjects will receive 4 repeated dose administrations, once every second week, and will after the fourth and last dose be followed for another 16 weeks. Each dose cohort, both in the SD and MD dose-escalation phases, will consist of four subjects (3 subjects in active treatment and 1 subject in placebo treatment). Subjects can only participate once in the trial and can not continue from the SD to the MD dose-escalation phase.

Upon completion of the SD and MD, dose-escalation phase, the decision to commence an MD expansion phase, at a selected dose level in 39 subjects (26 subjects in active treatment and 13 in placebo treatment), will be taken, in order to obtain Proof of Principle in a controlled setting. The selected dose level for the MD expansion phase will be the maximum tolerated dose, as observed from the two previous phases. The MD expansion phase will be identical to the MD dose-escalation phase design with regard to number of repeated doses, dosing interval, and visits. Subjects are not allowed to continue from the dose-escalation phases to the MD expansion phase.

The trial population will consist of males and females of non-childbearing potential, with moderate-to-severe stable plaque psoriasis, including subjects with psoriatic arthritis, aged between 18 and 75 years.

Primary objective:

<u>SD and MD dose-escalation phases</u>

1. To assess the safety and tolerability of SD and MD of Anti-IL-20 (109-0012) 100 mg/vial, administered subcutaneously, at escalating dose levels.

<u>MD expansion phase</u>

1. To assess the preliminary efficacy (Psoriasis Area and Severity Index (PASI), Physician´s Global Assessment (PGA), as well as Visual Analogue Scale (VAS) in subjects with psoriatic arthritis) of Anti-IL-20 (109-0012) 100 mg/vial at the maximum tolerated dose.

Secondary objectives:

SD and MD dose-escalation phases

1. To assess the preliminary efficacy (PASI, PGA and VAS in subjects with psoriatic arthritis) of Anti-IL-20 (109-0012) 100 mg/vial.
2. To assess the PK after dosing of Anti-IL-20 (109-0012) 100 mg/vial as SD and MD.
3. To explore PD and PG biomarkers of Anti-IL-20 (109-0012) 100 mg/vial and its effects on skin, vasculature, and the immune system.
4. To assess the immunogenicity of Anti-IL-20 (109-0012) 100 mg/vial (for the SD dose-escalation phase samples will only be analysed in case of safety concerns).

MD expansion phase

1. To assess the safety and tolerability of Anti-IL-20 (109-0012) 100 mg/vial.
2. To assess the PK after dosing of Anti-IL-20 (109-0012) 100 mg/vial as MD.
3. To explore PD and PG biomarkers of Anti-IL-20 (109-0012) 100 mg/vial and its effects on skin, vasculature, and the immune system.
4. To assess the immunogenicity of Anti-IL-20 (109-0012) 100 mg/vial.

Trial design:

This is an adaptive, randomised, double-blind, placebo-controlled, SD and MD, dose-escalation, safety and tolerability phase 1/2a, multi-centre trial, followed by an MD expansion phase at one selected dose level.

SD, dose-escalation phase

For the SD, dose-escalation phase, 24 subjects are planned to be included, assuming that six different dose levels will be completed. Each dose cohort will consist of four subjects; three receiving trial drug and one receiving placebo (3+1). Maximally, one additional dose cohort (3+1) can be added to each dose level, according to the dose-escalation rules, resulting in a total of 8 subjects at that specific dose level. The Study Safety Group will, based on review of safety data for each dose level, recommend if dose-escalation can take place (see12.6). The Study Safety Group will also have the mandate to add another cohort other than for safety reasons, if relevant.

MD, dose-escalation phase

In the MD, dose-escalation phase, five different dose levels are planned to be investigated. Similar to the SD phase, each dose level cohort will consist of four subjects (3+1). This means that 20 subjects are planned to be included, with the possibility to add maximally one extra dose cohort at each dose level, according to the dose-escalation rules. The Study Safety Group will, based on review of safety data for each dose level, recommend if dose-escalation can take place (see 12.6). The Study Safety Group will also have the mandate to add another cohort other than for safety reasons, if relevant. The Study Safety Group will have evaluated three dose levels in the SD phase before initiation of the MD dose-escalation phase.

MD, expansion phase

The MD expansion phase will be initiated as long as there is an acceptable safety profile and an indication of efficacy. There will also be the possibility to go directly into a phase 2 dose-finding trial, and refrain from the MD expansion phase. The decision whether or not to continue with the MD expansion phase will be taken based on unblinded safety and efficacy data. Subjects included in the MD expansion phase will be treated at one single dose level of Anti-IL-20 (109-0012) 100 mg/vial, and in total 39 subjects will be included; 26 subjects will receive active trial drug and 13 subjects will receive placebo (N= 26+13). The selected dose level for the MD expansion phase will be the maximum tolerated dose, as observed from the SD and MD dose-escalation phases.

Trial population:

The trial population will consist of males and females of non-childbearing potential, between 18 and 75 years of age, with stable plaque psoriasis for at least 6 months, a body surface area (BSA) involvement of at least 5% in the SD dose-escalation phase and 10% in the MD dose-escalation and MD expansion phases, and a PGA score of at least 3. Subjects also having psoriatic arthritis will not be excluded, although no other form of psoriasis, e.g. guttate, palm/plantar and erythrodermic, will be accepted.

In total, assuming all dose levels to be completed, including the MD expansion phase, 83 subjects will be entered into the trial, not taking into account the extra dose cohorts decided upon by the Study Safety Group.

Assessments:

Assessments will be performed regularly throughout the duration of the trial according to the flow charts (Table 2–1 and Table 2–2), and Section 8, Methods and Assessments, and the following data will be evaluated:

Primary assessment:

- Safety and tolerability

Secondary assessments:

- Efficacy (primary assessment for the MD expansion phase)
- Pharmacokinetics
- Pharmacodynamics
- Pharmacogenomics
- Immunogenicity

Trial products:

The active drug product, Anti-IL-20 (109-0012) 100 mg/vial, will be provided as freeze dried powder, which will be reconstituted with sterile water for injection to a concentration of 100 mg/mL. For the lowest doses the 100 mg/mL solution will be diluted to 10 or 1 mg/mL. The placebo solution, Placebo (109-0012) 0 mg/mL, will be provided as liquid. The placebo solution will also be used as diluent for the reconstituted active drug product. The freeze dried powder as well as the placebo solution should be kept at 2-8 °C. Both the trial drug and the placebo formulation will be administered subcutaneously, using standard syringes. Anti-IL-20 (109-0012) 100 mg/vial will be administered at the following doses (see also Table 5–2):

Single dose, dose-escalation phase: 0.01, 0.05, 0.20, 0.6, 1.5 and 3.0.

Multiple dose, dose-escalation phase: 0.05, 0.20, 0.5, 1.0 and 2.0.

2 Flow charts

Table 2–1 Flow chart for single dose, dose-escalation phase

Visit number		1	2	3	4	5	6	7[1]	8	9[1]	10	11
Timing: weeks		0	1		2	3	5	7	9	11	13	16
Timing: days		0	1-2	3	8	15	29	43[1]	57	71[1]	85	106
Window: days		-21 to -4					± 3	± 3	± 3	± 3	± 3	± 3
Subject related info/assessments												
Informed consent		x										
Gene consent		x										
In/exclusion criteria		x	x									
Randomisation			x									
Demography		x										
Concomitant illness		x										
Test for tuberculosis and hepatitis B and C		x										
Medical history[2]		x										
Concomitant medication		x	x	x	x	x	x	x	x	x	x	x
Body measurements		x	x[3]						x[3]			x[3]
Physical examination		x										x
Pregnancy test, if applicable			x									
Efficacy												
PK sampling			x[4]	x	x	x	x		x		x	x
BSA		x	x[5]			x			x		x	x
PGA		x	x[5]			x			x		x	x
PASI, VAS (if applicable)			x[5]			x			x		x	x
Safety												
Adverse events		x	x	x	x	x	x	x	x	x	x	x
Antibodies			x[6]						x			x
Biochemistry		x	x[7]	x	x	x	x		x		x	x
Haematology		x	x[7]	x	x	x	x		x		x	x
Hormones		x[8]	x[7]	x	x	x	x		x		x	x
Urinalysis		x	x[7]	x	x	x	x		x		x	x
ECG		x	x[9]		x	x			x			x
Vital signs (BP, pulse and temp.)		x	x[10]	x	x	x	x		x		x	x
Local tolerability at injection site			x	x	x	x	x		x		x	x
Other assessments												
Serum/plasma biomarkers			x[11]	x	x	x	x		x		x	x
Molecular biomarkers			x[12]		x	x						
Trial material												
Drug accountability			x									

Visit number		1	2	3	4	5	6	7[1]	8	9[1]	10	11
Timing: weeks		0	1		2	3	5	7	9	11	13	16
Timing: days		0	1-2	3	8	15	29	43[1]	57	71[1]	85	106
Window: days		-21 to -4					± 3	± 3	± 3	± 3	± 3	± 3
Subject related info/assessments												
Informed consent		x										
Administration of trial product			x									

[1]: Only telephone contact, no visit at site.

[2]: Including known history of human immunodeficiency virus (HIV).

[3]: Weight only.

[4]: PK sampling of 109-0012 will occur prior to dosing (0 hours) and at 2, 4, 8, 10 and 24 hours after drug administration.

[5]: Assessments will be performed prior to dosing on day 1, and 24 hours post-dosing.

[6]: Sampling will occur prior to dosing (0 hours).

[7]: Sampling will occur prior to dosing (0 hours) and 24 hours post-dosing.

[8]: Including follicle stimulating hormone (FSH), if applicable, at screening.

[9]: Electrocardiogram (ECG) will be recorded prior to dosing (0 hours) and at 2, 8 and 24 hours after drug administration.

[10]: Blood pressure will be measured prior to dosing (0 hours) and at 1, 2, 4, 8 and 24 hours after drug administration.

[11]: Serum and plasma samples will be collected prior to dosing (0 hours) on day 1, and at 10 hours and 24 hours post-dosing. Serum aliquots will be used for IL-20 and sCD25 analyses. Serum and plasma aliquots will be stored for further analyses, e.g. by multianalyte profiling.

[12]: Whole blood samples for gene expression (microarray and qRT-PCR) analyses will be collected prior to dosing (0 hours) on day 1 and 24 hours post-dosing.

Table 2–2 Flow chart for multiple dose, dose-escalation, and expansion phases

Visit number	1	2	3	4	5	6	7	8	9	10	11	12
Timing: weeks	0	1		2	3	5	7	9	11	15	19	22
Timing: days	0	1	2	3	15	29	43-44[1]	57	71	99	127	148
Window: days	-21 to -4							± 3	± 3	± 3	± 3	± 3
Subject related info/assessments												
Informed consent	x											
Gene consent	x											
In/exclusion criteria	x	x										
Randomisation		x										
Demography	x											
Concomitant illness	x											
Medical history[2]	x											
Test for tuberculosis and hepatitis B and C	x											
Concomitant medication	x	x	x	x	x	x	x	x	x	x	x	x
Body measurements	x	x[3]			x[3]	x[3]	x[3]					x[3]
Physical examination	x											x
Pregnancy test, if applicable		x										
Efficacy												
PK sampling		x[4]	x	x	x[5]	x[5]	x[4]	x	x	x	x	x
BSA	x	x[6]	x		x	x	x	x		x		x
PGA	x	x[6]	x		x	x	x	x		x		x
PASI, VAS (if applicable)		x[6]	x		x	x	x	x		x		x
Quality of life[13]		x						x				x
Safety												
Adverse events	x	x	x	x	x	x	x	x	x	x	x	x
Antibodies		x[7]					x[7]			x		x
Biochemistry	x	x[7]	x	x	x[7]	x[7]	x[7]	x	x	x	x	x
Haematology	x	x[7]	x	x	x[7]	x[7]	x[7]	x	x	x	x	x
Hormones	x[8]	x[7]	x	x	x[7]	x[7]	x[7]	x	x	x	x	x
Lipids[9]		x[7]						x				
Urinalysis	x	x[7]	x	x	x[7]	x[7]	x[7]	x	x	x	x	x
ECG	x	x[10]			x[10]	x[10]	x[10]	x				x
Vital signs (BP, pulse and temp.)	x	x[11]	x	x	x[11]	x[11]	x[11]	x	x	x	x	x
Local tolerability at injection site		x	x	x	x	x	x	x	x	x	x	x
Other Assessments												
Serum/plasma biomarkers		x[12]	x	x	x[12]	x[12]	x[12]	x	x	x	x	x
Biopsy (optional)		x[14,15]			x[14]					x[14]		
Genotyping[13]		x										

Visit number	1	2	3	4	5	6	7	8	9	10	11	12
Timing: weeks	0	1		2	3	5	7	9	11	15	19	22
Timing: days	0	1	2	3	15	29	43-44[1]	57	71	99	127	148
Window: days	-21 to -4							± 3	± 3	± 3	± 3	± 3
Subject related info/assessments												
Informed consent	x											
Molecular biomarkers		x[16]	x	x	x[16]							
Trial material												
IVRS call	x	x			x	x	x					
Drug accountability		x			x	x	x					
Administration of trial product		x			x	x	x					

[1]: A 24-hour PK sample after last drug administration should be collected at day 44 (no other assessments will be performed).

[2]: Including known history of HIV.

[3]: Weight only.

[4]: PK sampling of 109-0012 will be performed prior to dosing (0 hours) and at 1, 2, 4 and 24 hours after first and last drug administration.

[5]: Blood sampling for PK measurement of 109-0012 will be performed prior to drug administration (0 hours) in order to obtain a trough value.

[6]: Assessments will be performed prior to dosing.

[7]: Sampling will occur prior to dosing (0 hours).

[8]: Including FSH (if applicable) at screening.

[9]: Subjects must be fasting from midnight the same day until blood sampling.

[10]: ECG will be recorded prior to dosing (0 hours) and at 2 and 4 hours after dosing.

[11]: Blood pressure will be measured prior to dosing (0 hours) and at 1, 2 and 4 hours after dosing.

[12]: Serum and plasma samples will be collected prior to dosing (0 hours). Serum aliquots will be used for IL-20 and sCD25 analyses. Serum and plasma aliquots will be stored for further analyses, e.g. by multianalyte profiling

[13]: Only for MD expansion phase.

[14]: Lesional biopsies (1x6 mm) will be collected at Day 2, 15 and 99. At Day 2 and Day 15 this will occur prior to dosing (0 hours).

[15]: Non-lesional biopsy (1x3 mm) will be collected only at baseline.

[16]: Whole blood samples for gene expression (microarray and qRT-PCR) analyses will be collected prior to dosing (0 hours).

3 Introduction

In this document the Principal Investigator will be referred to as the Investigator.

3.1 Basic information

3.1.1 Psoriasis

Psoriasis is a T-cell mediated inflammatory disease where the activation of the immune system, via cytokines, results in epidermal hyperplasia, hyperproliferation of keratinocytes and cutaneous infiltration by granulocytes.

It is a serious, chronic disease, characterised by thickened erythematous plaques, usually located on elbows, knees, scalp, lower back, face, palms, and soles of the feet.[1] Psoriasis is associated with significant co-morbidity (obesity, cardiovascular morbidity) and with negative influence on the quality of life.

The prevalence of psoriasis is 1-3% and the mean age of onset is 28 years. Among different types, plaque psoriasis is the most common type, accounting for > 80% of subjects, and men and women are equally affected. Furthermore, 10-30% of subjects with psoriasis suffer from joint pain or arthritis. There is currently no cure for psoriasis, which affects about 125 million people worldwide. Approximately 25% of all patients experience moderate-to-severe disease symptoms.[2]

The aetiology of psoriasis is unknown, but genetic factors can determine the predisposition, while stress, skin injuries, infections, certain medications and sunburn can trigger the development of psoriasis. T lymphocytes, especially $CD8^+$ and $CD4^+$ cells, are involved in the mediation of the disease. $CD8^+$ and $CD4^+$ cells produce T_H1 cytokines, of which mainly interferon γ (IFNγ), interleukin-2 (IL-2) and tumour necrosis factor α (TNFα) play important roles for the pathogenesis of psoriasis. Furthermore, TNFα stimulates the production of other inflammatory cytokines and promotes angiogenesis, also observed in psoriasis. The initiation of psoriasis by T-cells is a complex process, and T-cells must first become activated before they can migrate to the skin and start secreting cytokines.[3-6] Moreover, serum levels of sCD25 (soluble form of the IL-2α receptor) which is secreted from T cells upon activation, has been shown to correlate to the PASI score during treatment.[7]

3.1.2 Existing treatment

There exists no permanent cure for psoriasis, and the available treatments aim at improving the quality of life for the subjects, and to reduce the extent and severity of the disease. The treatment depends mainly on the severity of the disease, but also on local practice. Topical treatment is primarily recommended for mild psoriasis, and vitamin D-analogues, such as calcipotriol, are commonly used, alone or in combination with local corticosteroid treatment.[8] Treatment of

moderate-to-severe psoriasis is often initiated with UVB (ultraviolet light B) radiation, or PUVA (UVA radiation together with systemic psoralene treatment). Systemic treatment for subjects with moderate to severe psoriasis, often involving arthritis, includes methotrexate (being the single most used systemic agent), cyclosporin and retinoids.[9]

Monoclonal antibodies, mostly humanised, belong to the more recently developed treatments, commonly called biologics. Treatment with TNFα-inhibitors (etanercept, adalimumab and infliximab) and T-cell inhibitors (alefacept and efalizumab) has become more and more common, although still only constituting less than 5% of the total usage of systemic agents.[10]

3.1.3 IL-20

IL-20 belongs to the IL-10 family, also including IL-19, IL-22, IL-24 and IL-26, IL-28A, IL-28B and IL-29, which have high secondary structural similarities. The amino acid identity between these cytokines is however low. Activated monocytes are the main production sites for IL-20, which exerts its action through two receptor dimers, i.e. IL-20R1/IL-20R2 (type I) and IL-22R1/IL-20R2 (type II). When these two receptor dimers aggregate, a signal transduction is initiated. Both the type I and type II receptor complexes have been detected in several tissues, such as epidermis, lungs, brain and glands [11, 12], but have so far not been detectable directly in immune cells. In lesional skin from psoriatic subjects both type I and type II are expressed.[11-14]

There is strong evidence that IL-20 is involved in the pathogenesis of psoriasis, and increased IL-20 messenger ribonucleic acid (mRNA) levels have been detected in lesional skin from psoriatic subjects, where the expression primarily is located to the basal and suprabasal keratinocytes. [13] Recently, it was demonstrated that IL-20 expression is induced by proinflammatory stimuli, i.e. IL-1β, IL-6 and UVB radiation, and it was also discovered that recombinant IL-20 stimulates T-cell proliferation in cell cultures of purified human CD45RA+ T-cells. Furthermore, IL-20 seems to be necessary for amplifying and sustaining keratinocyte activation.[15, 16]

Overexpression of IL-20 in transgenic mice have been interpreted as a psoriasis-like phenotype of the skin, though histologically not including the inflammatory component of human psoriasis.[14]

Other experiments performed in mice also clearly indicate IL-20 to be involved in the development of psoriasis. In a xenograft model where lesional psoriatic skin is transplanted on the back of SCID mice, Anti-IL-20 treatment with both polyclonal and monoclonal antibodies resulted in a partial resolution of psoriatic plaques, and a decrease in the semi-quantitative psoriasis scores compared to vehicle. Anti-IL-20 also prevented the induction of psoriasis in mice transplanted with non-lesional skin and injected with activated autologous peripheral blood mononuclear cells. Finally, treatment with recombinant hIL-20, induced psoriasis in transplanted non-lesional skin, injected with autologous non-activated PBMC (in-house data).

3.1.4 Trial drug

Novo Nordisk has produced a fully human Anti-IL-20 mAb, called 109-0012, which binds (K_D $3.1x10^{-11}$ M) to and neutralises the pharmacological activity of human IL-20. 109-0012 is an IgG_4 isotype, with high efficacy (EC_{50} $2.7x10^{-10}$), which is chemically and physically stable and does not induce complement activation. It is expressed in the CHO-K1-SV line using the Lonza Glutamine Synthetase expression system and no animal components are used during the production. Preliminary results from toxicology studies in non-human primates and mice have raised no safety concerns so far (please see IB,1st Edition and any updates hereof).

3.2 Rationale for the trial

The rationale for the use of 109-0012 in the treatment of psoriasis is based on several reports from the literature and from preclinical development (see 3.1.3).

At present there exists no permanent cure for psoriasis. Current treatment options only have moderate effect, although recently introduced biologics, especially TNF-α blockers, have shown improved sustained efficacy with PASI75 scores around 60%. In addition, there are also subjects who are refractory to available treatments or have a waning effect over time, as well as a rather delayed time of onset of clinical response. Furthermore, many drugs have significant side effects such as serious infections and potential malignancies. Thus, there is a need for developing a tolerable, safe, convenient, fast-acting and effective drug, which could offer a sustained remission to subjects suffering from psoriasis.

The trial will be conducted in adherence to "Guideline on Requirement for First-in-Man Clinical Trials for High Risk Medicinal Products, 2007" and "Guideline on Clinical Investigations of Medicinal Products Indicated for the Treatment of Psoriasis, 2004".[10, 17, 18]

4 Objectives and endpoints

4.1 Objectives

Primary objective:

SD and MD dose-escalation phases

1. To assess the safety and tolerability of SD and MD of Anti-IL-20 (109-0012) 100 mg/vial, administered subcutaneously, at escalating dose levels.

MD expansion phase

1. To assess the preliminary efficacy (PASI, PGA, as well as VAS in subjects with psoriatic arthritis) of Anti-IL-20 (109-0012) 100 mg/vial at the maximum tolerated dose.

Secondary objectives:

SD and MD dose-escalation phases

1. To assess the preliminary efficacy (PASI, PGA and VAS in subjects with psoriatic arthritis) of Anti-IL-20 (109-0012) 100 mg/vial.
2. To assess the PK after dosing of Anti-IL-20 (109-0012) 100 mg/vial as SD and MD.
3. To explore PD and PG biomarkers of Anti-IL-20 (109-0012) 100 mg/vial and its effects on skin, vasculature, and the immune system.
4. To assess the immunogenicity of Anti-IL-20 (109-0012) 100 mg/vial (for the SD dose-escalation phase samples will only be analysed in case of safety concerns).

MD expansion phase

1. To assess the safety and tolerability of Anti-IL-20 (109-0012) 100 mg/vial.
2. To assess the PK after dosing of Anti-IL-20 (109-0012) 100 mg/vial as MD.
3. To explore PD and PG biomarkers of Anti-IL-20 (109-0012) 100 mg/vial and its effects on skin, vasculature, and the immune system.
4. To assess the immunogenicity of Anti-IL-20 (109-0012) 100 mg/vial.

4.2 Endpoints

Primary endpoint:

SD and MD dose-escalation phases

1. The primary endpoint for safety will be observed toxicity assessed using the US National Cancer Institute common terminology criteria for adverse events version 3 (CTCAEv3). Dose limiting toxicities (DLT) will be recorded, and maximum tolerated dose (MTD) will be determined according to the criteria described in 12.1.1 and 12.1.2.

MD expansion phase

- The primary endpoint for efficacy is PASI (PASI75).

Secondary endpoints:

SD and MD dose-escalation phases

1. Efficacy
    - PGA
    - PASI (PASI75)
    - For subjects with psoriatic arthritis, assessment of arthritis symptoms using VAS

2. Pharmacokinetics
    - Terminal serum half-life ($t_{½}$)
    - Maximum observed serum concentration (C_{max})
    - Time to reach maximum serum concentration (t_{max})
    - Accumulation index (R_{acc})
    - Area under the serum concentration-time curve ($AUC_{0-\tau}$, AUC_{0-t} and AUC)

3. Pharmacodynamic biomarker assessments
    - Serum levels of IL-20 and sCD25
    - Proteomic profiling of serum and plasma biomarkers, e.g. by multi-analyte profiling

4. Pharmacogenomic biomarker assessment
    - Gene expression analyses in whole blood samples by global transcriptome profiling and quantifiable reverse transcriptase polymerase chain reaction (qRT-PCR) of selected biomarkers related to 109-0012 effects.

5. Immunogenicity assessment

- Detection of antibodies against 109-0012 and if present neutralising activity will be measured.

6. Safety

In addition to the primary endpoint the following endpoints will be recorded as secondary safety endpoints:

- Vital signs (blood pressure, pulse and temperature)
- ECG
- Laboratory safety parameters of haematology, biochemistry, hormones, lipids and urinalysis
- Immunogenicity (antibodies to 109-0012)
- Physical examination

Pharmacodynamic and pharmacogenomic parameters will not be analysed during the ongoing trial.

<u>MD expansion phase</u>

1. Safety will be evaluated and observed toxicity will be assessed using the US National Cancer Institute CTCAE version 3. Furthermore the following parameters will also be assessed:
   - Vital signs (blood pressure, pulse and temperature)
   - ECG
   - Laboratory safety parameters of haematology, biochemistry, hormones, lipids and urinalysis
   - Immunogenicity (antibodies to 109-0012)
   - Physical examination

2. The following parameters will be recorded as secondary efficacy endpoints:
   - PGA
   - For subjects with psoriatic arthritis, assessment of arthritis symptoms using VAS
   - Quality of life (QoL; DLQI)

3. Pharmacokinetics
   - Terminal serum half-life ($t_{½}$)
   - Maximum serum concentration (C_{max})
   - Time to reach maximum serum concentration (t_{max})
   - Accumulation index (R_{acc})
   - Area under the concentration-time curve ($AUC_{0-τ}$, AUC_{0-t} and AUC)

4. Pharmacodynamic biomarker assessments

a) Serum/plasma samples:

- Serum levels of IL-20 and sCD25
- Proteomic profiling of serum and plasma biomarkers, e.g. by multi-analyte profiling

b) Skin biopsies:

- Histopathological scoring including assessment of epidermal thickness and proliferation, e.g. by hematoxylin & eosin (H&E), keratin 16, and Ki67 stain
- Exploratory assessment of 109-0012 effects on skin, vasculature, and immune system may include but not be limited to:
  - expression of IL-20 and IL-20 receptors
  - infiltrating leukocyte subsets, e.g. CD3, CD11c, and CD68
  - IL-20 signalling, e.g. phosphorylated signal transducer and activator of transcription 3 (pSTAT3)
  - angiogenesis, e.g. CD31 stain

Lesional 6 mm biopsies will be collected. Each biopsy will be split into two halves for cryo preservation.

5. Pharmacogenomic biomarker assessments

a) Blood samples:

- Gene expression analysis by global transcriptome profiling and qRT-PCR of selected biomarkers related to 109-0012 effects.
- Genotyping of IL-20 single nucelotide polymorphisms (SNP) haplotypes.

b) Skin biopsies:

- Gene expression analysis by global transcriptome profiling and qRT-PCR of selected biomarkers related to psoriasis and 109-0012 effects.

Lesional 3 mm skin biopsies will be collected and cryo preserved.

6. Immunogenicity assessment
   - Detection of antibodies against 109-0012 , and if present neutralising activity will be measured.

7. QoL assessed by DLQI

Pharmacodynamic and pharmacogenomic parameters will not be analysed during the ongoing trial.

5 Trial design

5.1 Type of trial

This is an adaptive, randomised, multicentre, double-blind, placebo-controlled phase 1/2a trial, studying the safety and tolerability of different dose levels of SD and MD of Anti-IL-20 (109-0012) 100 mg/vial. The trial will be performed in subjects with moderate to severe stable plaque psoriasis, with or without psoriatic arthritis. The MD dose-escalation will be performed partly in parallel with the SD dose-escalation phase; the first dosing of the MD dose-escalation phase will start when the third dose level of the SD dose-escalation phase has been evaluated by the Study Safety Group.

Initiation of the MD expansion phase, at the maximum tolerated dose, will depend on results from the dose-escalation phases and it will only be initiated as long as there is an acceptable safety profile. There will also be the possibility to go directly into a phase 2 dose-finding trial, and refrain from the MD expansion phase. The decision whether or not to continue with the MD expansion phase will be taken based on unblinded safety and efficacy data.

SD, dose-escalation phase

A total of 24 subjects (18 receiving active treatment and 6 receiving placebo) will be included in the SD dose-escalation phase. Each of the six different dose cohorts (maximum dose of 3 mg/kg) will consist of four subjects; three receiving active treatment and one receiving placebo. Subjects will be followed until trial Week 16.

MD, dose-escalation phase and MD expansion phase

A total of 20 subjects (15 receiving active treatment and 5 receiving placebo) will be included in the MD dose-escalation phase. Each of the five different dose cohorts (maximum dose of 2 mg/kg) will consist of four subjects; three receiving active treatment and one receiving placebo.

In the MD expansion phase, a total of 39 subjects (26 receiving active treatment and 13 receiving placebo) will be included, at the maximum tolerated dose. Subjects are not allowed to have participated in the previous phases (SD and MD dose-escalation phases) of the trial. Number of visits, time points for visits, dosing intervals and number of repeated doses will be identical between the MD dose-escalation phase and the MD expansion phase. See Section 8.1.2.

During both the MD dose-escalation phase and the MD expansion phase, subjects will receive four repeated trial drug administrations (once every second week) and will be followed through trial Week 22.

Time schedules

The overall time schedules and the trial design is shown in

Figure 5–1. The expected trial duration for the SD and MD dose-escalation phase is approximately 19 months (77 weeks), and 9 months for the MD expansion phase.

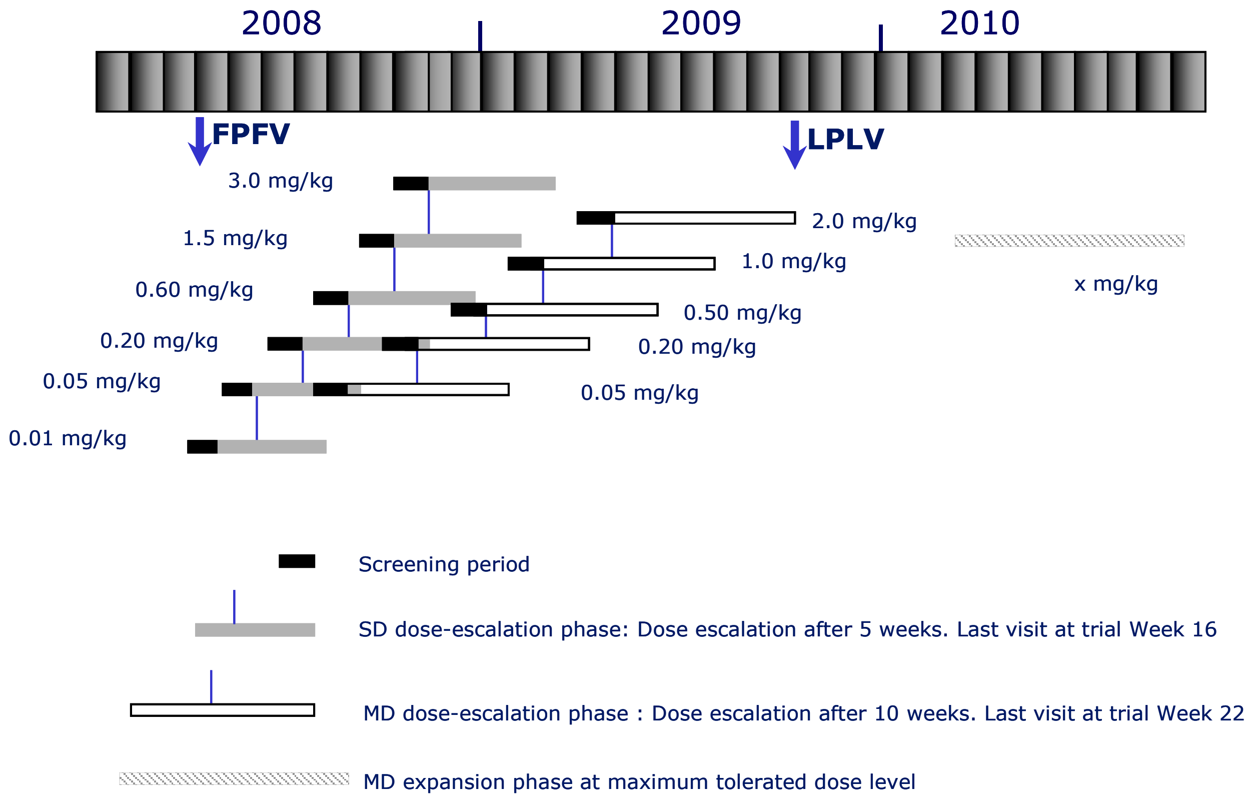

Figure 5–1 : Illustration of the concept of the FHD trial, showing the SD phase at six dose levels, the MD phase at five dose levels, and an MD expansion phase at one dose level.

5.2 Rationale for trial design

A cautious approach is recommendable when starting FHD trials with biologics. If feasible, the dose should be selected based on calculations using the “minimal anticipated biological effect level (MABEL)” approach. Due to lack of preclinical in vivo models and predictive in vitro models this has not been possible. However, a low and presumed safe first single dose has been rationalised from the lack of toxicity in the toxicology studies at all dose levels, combined with simulated human pharmacokinetics of 109-0012 and resulting IL-20 serum concentrations.

All subjects in each dose cohort will be randomised and there will be a time interval of at least 24 hours between dosing of all subjects in each dose cohort of the SD dose-escalation phase. Subjects will be monitored at the research facility during the first 24 hours. Before continuing to the next dose level, safety data for all subjects through trial Day 15 as well as PK data for all subjects through trial Day 15 will be evaluated by the Study Safety Group (See Section 12.6).

All four subjects in each dose cohort can be dosed on the same day during the MD dose-escalation phase. During the MD dose-escalation phase subjects will be monitored continuously for the first 8 hours after the first drug administration. The second dose should be administered on trial Day 15, the third dose on trial Day 29, and the fourth and last dose will be given on trial Day 43. Subjects will be monitored in-house at the research facility for 4 hours after the second, third and fourth dose.

The dose-escalation decision in the MD dose-escalation phase will be made based on safety data from all subjects obtained through trial Day 57, two weeks after the last dose, as well as PK data through trial Day 57. Table 5–1 shows the proposed doses and dose intervals for the dose-escalation phases and the relative time points for starting each cohort.

The MD dose-escalation phase will be performed in parallel with the SD phase, but the first dose cohort of the MD dose-escalation phase will not commence until the third dose cohort of the SD phase has been evaluated by the Study Safety Group (Table 5–1). This means that the first accumulated multiple dose has been covered by the single doses already administered. This trial design is considered both safe and efficient.

This adaptive trial design has been chosen in order to increase the chances of finding the optimal dose level as early as possible, to save as much time as possible and not to use more patients than really needed.

Placebo will be used as control in order to distinguish any potential effects between the drug itself and other effects, not related to the trial drug itself. Furthermore, due to the fluctuating character of the disease, placebo is needed in order to ascertain sensitivity.

5.3 Treatment of subjects

Anti-IL-20 (109-0012) 100 mg/vial or Placebo (109-0012) 0 mg/mL will be administered subcutaneously into the abdomen using a standard syringe. The active doses will range from 0.01 mg/kg to 3 mg/kg in the SD phase and from 0.05 mg/kg to 2 mg/kg in the MD phase. The maximum dose of 3 mg/kg has been chosen due to injection volume limitations. The dose to be investigated in the MD expansion phase will be the maximum tolerated dose reached in the previous phases and will not commence until the SD and MD dose-escalation phases have been

completed. For more details see Table 5–1, which presents the anticipated dosing schedule for the different dose cohorts for the SD and MD dose-escalation phases.

The freeze dried drug product will be dissolved in Water for Injection at a concentration of 100 mg/mL and further diluted with the placebo formulation to 10 or 1 mg/mL for the lowest doses. The volume of each injection will not exceed 1.2 mL. The placebo formulation will be provided as a liquid formulation and will have an identical appearance as the reconstituted freeze dried active drug at the lowest doses. At the higher doses the reconstituted active drug solution might differ in colour due to high protein content.

Table 5–1 Dose levels for the SD and MD dose-escalation phases and proposed initiation times.

Time when the different dose levels will be started (weeks)	SD, mg/kg	MD, mg/kg (accumulated doses after four administrations)
**FPFV (W 0) to LPFV (W 55)**	**Maximum dose level** 3 mg/kg	**Maximum dose level** 2 mg/kg
W* 0	Level 1: 0.01	
W 5	Level 2: 0.05	
W 10	Level 3: 0.20	
W 15	Level 4: 0.6	Level 1: 0.05 (0.2)
W 20	Level 5: 1.5	
W 25	Level 6: 3.0	Level 2: 0.20 (0.8)
W 35		Level 3: 0.5 (2.0)
W 45		Level 4: 1.0 (4.0)
W 55		Level 5: 2.0 (8.0)
**LPLV:** W 77		

* Related to the dosing of the first subject in the trial, and not to trial Week of each individual subject.

Dosing will occur every second week during the MD phases (dose-escalation and expansion phase), for a total of four doses. All subjects will be followed for 16 weeks after last dose and with a

suggested half-life of about 15-20 days the drug is then anticipated to have been sufficiently cleared from the body.

Other anti-psoriatic treatments, except moisturising cream and low potency corticosteroid cream (group 1), will not be allowed during the trial. For more specific details regarding prohibited concomitant medication see section 6.3.

5.4 Rationale for treatment

The trial drug is a protein and can therefore not be administered orally. A subcutaneous injection in the abdomen has been chosen as the most appropriate administration. During the trial, medical personnel will perform the injection. Due to risk for unblinding, the person performing the injection must be trial independent and not take part in any other trial related activities.

The first dose has been selected based on results from pre-clinical studies and biomodelling/simulation data. The expected dose range is between 0.01 mg/kg and 3 mg/ kg during the SD phase, and between 0.05 mg/kg and 2 mg/kg during the MD dose-escalation phase. Initiation of the MD expansion phase, at the maximum tolerated dose, will depend on results from the dose-escalation phases and will be decided upon when the SD and MD dose-escalation phases have been completed. It will only be initiated as long as there is an acceptable safety profile. There will also be the possibility to go directly into a phase 2 dose-finding trial, and refrain from the MD expansion phase, provided there is an acceptable effect. The decision whether or not to continue with the MD expansion phase will be taken based on unblinded safety and efficacy data.

During the MD dose-escalation phase and the expansion phase four repeated doses, once every second week, will be investigated. This dosing interval is chosen with respect to the expected half-life of the trial drug and future dosing convenience for subjects.

The decision for dose-escalation will be taken by the Study Safety Group according to the instructions in Table 5–2. Administration of Anti-IL-20 (109-0012) 100 mg/vial at the next dose level can only be initiated when the following criteria are met:

- Safety assessments and evaluations by the Study Safety Group through trial Day 15 for individual subjects in the current dose cohort of the SD dose-escalation phase, and through trial Day 57 of the MD dose-escalation phase.
- Evaluation of PK data by the Study Safety Group through trial Day 15 for individual subjects in the current dose cohort of the SD dose-escalation phase, and through trial Day 57 of the MD dose-escalation phase.

- The next dose level has been approved by the Study Safety Group. The Study Safety Group will also have the mandate to add another cohort other than for safety reasons, if relevant.

Table 5–2 Dose-escalation rules

Number of subjects with grade 3 toxicity (CTCAE) related to trial product administration	Decision rules
0	Enter four subjects (3+1) at the next dose level, according to Table 5–1.
1 of 4	Enter four subjects (3+1) at the same dose level as the current level where the toxicity event occurred.
1 of 8	Enter four subjects (3+1) at the next dose level, according to Table 5–1.
≥2	The dose-escalation will be stopped at this level, and no further increase of dose will occur. There will however be the possibility to add four (3+1) subjects at one or maximally two different dose levels between the current dose limiting level and the previous dose level.<br>• The maximum tolerated dose (MTD) is the highest dose level below the maximum administered dose (MAD) where none or 1 subjects experience a DLT.<br>• The maximum administered dose is the highest dose given in the trial.<br>• The maximal feasible dose is 3 mg/kg.

6 Trial population

6.1 Number of subjects to be studied

Country planned to participate: US

Planned number of subjects to be screened (i.e. documented informed consent): 120

Planned number of subjects to be randomised/started on trial product(s): 96

Planned number of subjects to complete the trial: 83

Anticipated number of trial sites: 5-10

Anticipated number of subjects to be randomised/started on trial product(s) at each trial site: 24 for the SD dose-escalation phase (one single site will be used for this phase only) and 4-15 for the MD dose-escalation and MD expansion phase.

6.2 Inclusion criteria

1. Informed consent obtained before any trial-related activities. (Trial-related activities are any procedure that would not have been performed during normal management of the subject).
2. Subject with moderate to severe stable chronic plaque psoriasis
    - Duration for at least 6 months
    - With or without psoriatic arthritis
3. BSA ≥5% (for SD phase), BSA ≥10% (for MD dose-escalation and expansion phase)
4. PGA ≥3
5. If a male subject, he must agree to use effective method of birth control (condom with spermicide)
6. If a female subject, she must be postmenopausal (postmenopausal for at least 1 year, otherwise FSH-levels ≥ 40 mIU/mL) or of non-childbearing potential
7. Age between 18 and 75 years, inclusive
8. BMI ≤ 38.0 kg/m^2

6.3 Exclusion criteria

1. Known or suspected allergy to trial product(s) or related products
2. Previous participation in this trial, i.e. randomised subjects
3. Receipt of any investigational drug within 5 half-lives of the drug prior to this trial
4. Concomitant anti-psoriatic treatment
    - Etanercept within the last month

- Infliximab, efalizumab and alefacept within the 2 last months
- Adalimumab within the last 3.5 months
- Cyclosporine, acitretin, and methotrexate within the last month
- UV-B within the last 2 weeks
- PUVA within the last month
- Topical treatment within the last 2 weeks, except moisturising cream (will be supplied by NN) and mild corticosteroid cream of group 1

5. Any of the following concomitant medication within the last 2 weeks:
    - Immunosuppressants
    - Disease modifying anti-rheumatic drugs (DMARDs), including anti-malaria drugs
    - Interferon
    - Lithium
    - Selective cyclooxygenase-2 (COX-2) inhibitors

6. Other forms of psoriasis
    - Pure palm/plantar psoriasis
    - Pustular psoriasis
    - Guttate psoriasis
    - Erythrodermic psoriasis
    - Unstable psoriasis

7. Active or latent tuberculosis (determined by test)
8. Infectious disease requiring systemic anti-infectious treatment within the 2 weeks prior to administration of trial drug
9. Known history of HIV
10. Hepatitis B and/or C (determined by test)
11. Live virus or bacteria vaccines within the last month before drug administration
12. Known active herpes/herpes zoster/cold sores
13. Renal insufficiency: Serum creatinine ≥ 1.5x upper limit of normal (ULN)
14. Hepatic insufficiency: ALT or AST ≥ 2.5x ULN (retesting is permitted within one week if first test is elevated)
15. Lymphoproliferative disease
16. History or signs of malignancy within the last 5 years (except fully excised basal cell or squamous cell carcinoma)
17. Subject with 5 or more basal cell or squamous cell carcinoma within the last 12 months
18. History of or current drug and/or alcohol abuse
19. Blood donation within the last 3 months (≥ 0.45 L)

20. Any other disease or clinically significant abnormality in laboratory parameters which, according to the investigator, might compromise the safety of the subject or interfere with participation in the trial or compromise the trial objective

6.4 Withdrawal criteria

The subject may withdraw at will at any time.

The subject may be withdrawn from the trial at the discretion of the Investigator or the sponsor due to safety concerns, or if judged non-compliant with trial procedures.

A subject must be withdrawn if the following applies:

1. Non-compliance with protocol procedures
2. Drug limiting toxicity (DLT) or other serious or significant adverse event that warrants discontinuation in the opinion of the investigator
3. Sponsor closure of the trial
4. Withdrawal of informed consent

For the MD dose-escalation phase and the MD expansion phase an **IVRS call** must be performed if the subject is withdrawn.

6.5 Subject replacement

Subjects who drop out or are withdrawn before completion of trial Day 29 in the SD phase and trial Day 57 in the MD dose-escalation and expansion phases, should be replaced. For the SD and MD dose-escalation phases replacement must occur to the same dose cohort as well as the same treatment.

6.6 Rationale for trial population

The scientific rationale for including subjects with psoriasis rather than healthy subjects is based on the fact that the level of IL-20 in healthy non-lesional skin is limited. Therefore, the relevance of the tolerability data obtained in healthy subjects may be questioned and potential pharmacologically induced adverse events may also not be revealed. Furthermore, subjects in clinical trials of therapeutic and diagnostic products, including mAbs, should be representative of the population targeted for eventual product use. By using psoriatic subjects there will also be the possibility to obtain an early clinical read-out, which would not be possible if using healthy volunteers.

Women of child bearing potential are not allowed to participate and males must accept the use of contraception since no preclinical reproduction toxicity studies have yet been performed.

Appropriate wash-out periods (5 half-lives) must be applied for other systemic anti-psoriatic treatments since they might otherwise confound the results.

Psoriatic subjects generally have a higher risk of developing lymphoproliferative diseases and malignancies and previous treatments might also increase the risk. Carcinomas, lymphoproliferative diseases and tuberculosis are therefore listed as exclusion criteria in order not to confound the results.[19, 20]

7 Trial schedule

If consent is obtained and subjects are considered eligible they will be scheduled for a dosing visit, which is set to trial Day 1 for each subject, within 4 to 21 days after the screening visit.

There will be a recruitment period of approximately three weeks before each new cohort. A pre-screening contact can be established where subjects are informed about the trial and the criteria for participation.

Planned first trial site ready: 1 April 2008

Planned date for first patient first visit (FPFV): 7 April 2008

Planned completion of the last patient: November 2010

The end of the clinical trial is defined as the last visit of the last patient.

Planned completion of clinical trial report: April 2011

This trial is subject to registration before subject enrolment according to the specifications from the International Committee of Medical Journal Editors (ICMJE) [21]. Novo Nordisk will ensure the trial is registered at ClinicalTrials.gov according to the requirements from the Food and Drug Administration (FDA) [22] and ICMJE [21].

8 Methods and assessments

8.1 Visit procedures

8.1.1 Visit procedures for SD dose-escalation phase

Day -21 to Day -4 – screening

Subjects will be informed about the trial both verbally and written, and given ample time for questions and consideration. Informed consent must be obtained prior to any trial related activities. Thereafter, subjects will be provided with a subject number. The screening visit should take place 4 to 21 days prior to the first dosing visit. If necessary, the screening visit can be rescheduled within a week without collecting new urine and blood samples. If no dose cohort is currently open within 21 days after screening, a subject may be re-screened under a new subject number in order to be able to enter the trial at a later stage.

The Investigator must keep a subject screening and enrolment log.

The following data and samples will be collected and recorded at the screening visit (see also Table 2–1 in Section 2):

1. Signed and dated informed consent (before any trial related activities take place)
2. Gene consent
3. Demography
4. Body measurements
5. Medical history (including known history of HIV)
6. Physical examination
7. Vital signs
8. Test for hepatitis B and C
9. Test for tuberculosis
10. Adverse events
11. ECG
12. Haematology
13. Biochemistry
14. Urinalysis
15. Hormones (including FSH levels if applicable)
16. History of drug and/or alcohol abuse
17. BSA
18. PGA
19. Inclusion and exclusion criteria
20. Concomitant medication

21. Concomitant illness

Day 1 to Day 2 - dose administration

Results from the **screening** safety laboratory assessments must be available at Day 1 (dosing) visit. Subjects will come to the site in the morning for an over-night stay, and **before trial drug administration**, the following data and samples will be collected and recorded:

1. Vital signs
2. Body measurements (weight only)
3. ECG
4. BSA
5. PGA
6. PASI and VAS (if applicable)
7. Haematology
8. Biochemistry
9. Urinalysis
10. Hormones
11. Sampling for molecular biomarkers (baseline)
12. Sampling for serum/plasma biomarkers (baseline)
13. Sampling for antibodies (baseline)
14. Urine pregnancy test (if applicable)
15. Inclusion and exclusion criteria
16. Concomitant medication
17. Adverse events
18. PK sample (0 hours)

Thereafter subjects will be randomised to the lowest number available and receive a subcutaneous injection of either active treatment or placebo into the abdomen. Since the trial is double-blind, neither the subject nor the investigator/study nurse will know whether active treatment or placebo is administered. There is a 3:1 chance of receiving active treatment.

Samplings and recordings occurring after trial product administration:

1. Samples for PK measurements of 109-0012 will be collected at 2, 4, 8, 10 and 24 hours (Day 2) after drug administration.
2. ECG recordings will be performed at: 2, 8 and 24 hours (Day 2) after drug administration.
3. Blood pressure will be measured at: 1, 2, 4, 8 and 24 hours (Day 2) after drug administration.
4. Sampling for serum/plasma biomarkers will be performed at 10 hours and at 24 hours (Day 2) after drug administration.

5. BSA
6. PGA
7. PASI and VAS (if applicable)
8. Sampling for molecular biomarkers will be performed at 24 hours (Day 2) after drug administration.
9. Blood samples for haematology, biochemistry, and hormones will be collected, together with a urine sample, at 24 hours after drug administration.
10. Subjects will be closely monitored for the first 24 hours and any adverse events and/or local irritation at injection site will be registered.
11. Drug accountability will be performed.

After the 24-hour samples have been collected, ECG recording has been performed and blood pressure has been measured, the subject can leave the site.

<u>Day 3 to Day 85</u>

Subjects will come for regular visits at Day 3, 8, 15, 29, 57 and 85. From Day 29 a deviation of ± 3 days from the scheduled day will be allowed.

The following data and samples will be collected and recorded at **all visits to site**:

1. Vital signs
2. Haematology
3. Biochemistry
4. Urinalysis
5. Hormones
6. Serum/plasma biomarkers
7. Adverse events
8. Local tolerability
9. Concomitant medication
10. Collection of PK samples

Procedures which are not performed at every visit are listed below, together with the time point at which they should be performed:

<u>Day 8:</u> ECG and sampling for molecular biomarkers.

<u>Day 15:</u> ECG, sampling for molecular biomarkers, BSA, PGA, PASI, and VAS (if applicable).

Day 43 (± 3 days): The investigator/study nurse will call all subjects and check their status only (withdrawal criteria, concomitant medication, and adverse events).

Day 57 (± 3 days): ECG, BSA, PGA, PASI, VAS (if applicable), sampling for antibodies and body measurements (weight only).

Day 71 (± 3 days): The investigator/study nurse will call all subjects and check their status only (withdrawal criteria, concomitant medication, and adverse events).

Day 85 (± 3 days): BSA, PGA, PASI, VAS (if applicable).

Day 106 (± 3) – last visit

When subjects come for the last visit at Day 106 (± 3 days) after drug administration the following data and samples will be collected and recorded:

1. Body measurements (weight only)
2. Physical examination
3. Vital signs
4. Haematology
5. Biochemistry
6. Urinalysis
7. Hormones
8. ECG
9. BSA
10. PGA
11. PASI and VAS (if applicable)
12. Sampling for serum/plasma biomarkers
13. Sampling for antibodies
14. Concomitant medication
15. Local tolerability
16. Adverse events
17. PK sample

8.1.2 Visit procedures for MD dose-escalation phase

Day -21 to Day -4 – screening

Subjects will be informed about the trial both verbally and written, and given ample time for questions and consideration. Informed consent must be obtained prior to any trial related activities. Thereafter, subjects will be provided with a subject number through IVRS call. The screening visit

should take place within 4 to 21 days prior to the first dosing visit. If necessary, the screening visit can be rescheduled within a week without collecting new urine and blood samples. If no dose cohort is currently open within 21 days after screening, a subject may be re-screened under a new subject number in order to be able to enter the trial at a later stage.

The Investigator must keep a subject screening and enrolment log.

The following data and samples will be collected and recorded at the screening visit (see also Table 2–2 in Section 2):

1. Signed and dated informed consent (before any trial related activities are taking place)
2. Gene consent
3. Demography
4. Body measurements
5. Medical history (including known history of HIV)
6. Physical examination
7. Vital signs
8. Test for hepatitis B and C
9. Test for tuberculosis
10. Adverse events
11. ECG
12. Haematology
13. Biochemistry
14. Urinalysis
15. Hormones (including FSH levels if applicable)
16. History of drug and/or alcohol abuse
17. BSA
18. PGA
19. Inclusion and exclusion criteria
20. Concomitant medication
21. Concomitant illness
22. Register subject as screened in IVRS

Day 1 – first dose administration

Results from the **screening** safety laboratory assessments must be available at Day 1 (dosing) visit. At trial Day 1 all subjects must have been **fasting since midnight the same day** (only water will be allowed from midnight until collection of blood samples). The subject will come to the site in the

morning **and before trial drug administration**, the following data and samples will be collected and recorded:

1. Vital signs
2. Body measurements (weight only)
3. ECG
4. BSA
5. PGA
6. Haematology
7. Biochemistry
8. Urinalysis
9. Hormones
10. Lipids
11. PASI and VAS (if applicable)
12. Sampling for molecular biomarkers (baseline)
13. Sampling for serum/plasma biomarkers (baseline)
14. Sampling for antibodies (baseline)
15. Lesional skin biopsies (1x6 mm to be split for histopathology+IHC and array/qRT-PCR analyses) and non-lesional skin biopsies (1x3 mm for baseline histopathology+IHC). This procedure is optional.
16. Urine pregnancy test (if applicable)
17. Inclusion and exclusion criteria
18. Concomitant medication
19. Adverse events
20. PK sample (0 hours)

Thereafter subjects will be randomised **by IVRS** and receive a subcutaneous injection of either active treatment or placebo into the abdomen. Since the trial is double-blind, neither the subject nor the investigator/study nurse will know whether active treatment or placebo is administered. There is a 3:1 chance of receiving active treatment.

Samplings and recordings occurring after trial product administration:

1. Samples for PK measurement of 109-0012 will be collected at: 1, 2, 4 and 24 hours (Day 2) after drug administration.
2. Blood pressure will be measured 1, 2 and 4 hours after drug administration.
3. ECG will be recorded at 2 and 4 hours after drug administration.
4. Subjects will be closely monitored during their 8-hour-stay, and any adverse events and/or local irritation at injections site will be registered.

5. When all recordings have been finished, subjects are allowed to leave the site, but not before 8 hours after drug administration.
6. Drug accountability will be performed via **IVRS**.

<u>Day 2 and Day 3</u>

Subjects will return to site the day after drug administration as well as Day 3. The following data and samples will be recorded and collected:

1. Vital signs
2. Haematology
3. Biochemistry
4. Urinalysis
5. Hormones
6. Sampling for molecular biomarkers
7. Sampling for serum/plasma biomarkers
8. Concomitant medication
9. Local tolerability
10. Adverse events and toxicities
11. PK sample

PGA, BSA, PASI and VAS (if applicable) will only be assessed at Day 2.

<u>Day 15, Day 29 and Day 43 - dose administrations</u>

Subjects will come for drug administration at Day 15, day 29 and day 43. **Prior to trial product administration** the following data and samples should be recorded and collected:

1. Vital signs
2. Body measurements (weight only)
3. ECG
4. Haematology
5. Biochemistry
6. Urinalysis
7. Hormones
8. BSA
9. PGA
10. PASI and VAS (if applicable)
11. Sampling for serum/plasma biomarkers

12. Concomitant medication
13. Local tolerability
14. Adverse events
15. PK sample (through value)
16. IVRS dispensing call

Thereafter trial product administration will occur.

Samplings and recordings occurring after trial product administration:

1. ECG will be recorded at 2 and 4 hours after each drug administration.
2. Blood pressure will be measured at 2 and 4 hours after each drug administration.
3. After each drug administration subjects will be closely monitored during their 4-hour-stay.
4. Drug accountability will be performed via **IVRS.**

<u>Day 15:</u> Sampling for molecular biomarkers and lesional skin biopsies (1x6 mm to be split in half). The skin biopsy procedure is optional.

<u>Day 43:</u> Sampling for antibodies.

Subjects are allowed to leave the site 4 hours after drug administration when all samples have been collected and all assessments have been recorded.

<u>Day 44</u>

Subjects must return to site for a 24-hour PK sample after the last drug administration.

<u>Day 57 to Day 148 (± 3 days)</u>

Subjects will come for regular visits at Day 57, 71, 99, 127 and 148. From Day 57 and onwards a deviation of ± 3 days from the scheduled day will be allowed. The following data and samples will be recorded and collected at each of those visits:

1. Vital signs
2. Haematology
3. Biochemistry
4. Urinalysis
5. Hormones
6. Sampling for serum/plasma biomarkers
7. PK samples
8. Concomitant medication

9. Local tolerability
10. Adverse events

Procedures which are not performed at every visit are listed below together with the time point at which they should be performed:

Day 57 (± 3 days): Lipids, ECG, BSA, PGA, PASI and VAS (if applicable). At trial Day 57 all subjects must have been **fasting since midnight the same day** (only water will be allowed from midnight until collection of blood samples).

Day 99 (± 3 days): BSA, PGA, PASI and VAS (if applicable), and sampling for antibodies. Collection of lesional skin biopsies (1x6 mm to be split in half) is optional.

Day 148 (± 3 days): Body measurements (weight only), physical examination, ECG, BSA, PGA, PASI and VAS (if applicable), and sampling for antibodies.

8.1.3 Visit procedures for MD expansion phase

Day -21 to Day -4 – screening

Subjects will be informed about the trial both verbally and written, and given ample time for questions and consideration. Informed consent must be obtained prior to any trial related activities. Thereafter, subjects will be provided with a subject number through IVRS call. The screening visit should take place within 4 to 21 days prior to the first dosing visit. If necessary, the screening visit can be rescheduled within a week without collecting new urine and blood samples. If no dose cohort is currently open within 21 days after screening, a subject may be re-screened under a new subject number in order to be able to enter the trial at a later stage.

The Investigator must keep a subject screening and enrolment log.

The following data and samples will be collected and recorded at the screening visit (see also Table 2–2 in Section 2):

1. Signed and dated informed consent (before any trial related activities are taking place)
2. Gene consent
3. Demography
4. Body measurements
5. Medical history (including known history of HIV)
6. Physical examination
7. Vital signs
8. Test for hepatitis B and C
9. Test for tuberculosis

10. Adverse events
11. ECG
12. Haematology
13. Biochemistry
14. Urinalysis
15. Hormones (including FSH levels if applicable)
16. History of drug and/or alcohol abuse
17. BSA
18. PGA
19. Inclusion and exclusion criteria
20. Concomitant medication
21. Concomitant illness
22. Register subject as screened in IVRS

Day 1 – first dose administration

Results from the **screening** safety laboratory assessments must be available at Day 1 (dosing) visit. At trial Day 1 all subjects must have been **fasting since midnight the same** day (only water will be allowed from midnight until blood sample collection). The subject will come to the site in the morning **and before trial drug administration**, the following data and samples will be collected and recorded:

1. Vital signs
2. Body measurements (weight only)
3. ECG
4. BSA
5. PGA
6. PASI and VAS (if applicable)
7. Haematology
8. Biochemistry
9. Urinalysis
10. Hormones
11. Lipids
12. Sampling for molecular biomarkers (baseline)
13. Sampling for serum/plasma biomarkers (baseline)
14. Sampling for antibodies (baseline)
15. Blood sampling for genotyping

16. Lesional skin biopsies (1x6 mm to be split for histopathology+IHC and array/qRT-PCR analyses) and non-lesional skin biopsies (1x3 mm for baseline histopathology+IHC). This procedure is optional.
17. Urine pregnancy test (if applicable)
18. Inclusion and exclusion criteria
19. Quality of life questionnaire
20. Concomitant medication
21. Adverse events
22. PK sample (0 hours)

Thereafter subjects will be randomised **by IVRS** and receive a subcutaneous injection of either active treatment or placebo into the abdomen. Since the trial is double-blind, neither the subject nor the investigator/study nurse will know whether active treatment or placebo is administered. There is a 2:1 chance of receiving active treatment.

Samplings and recordings occurring after trial product administration:

1. Samples for PK measurement of 109-0012 will be collected at: 1, 2, 4 and 24 hours (Day 2) after drug administration.
2. Blood pressure will be measured 1, 2 and 4 hours after drug administration.
3. ECG will be recorded at 2 and 4 hours after drug administration.
4. Subjects will be closely monitored during their 8-hour-stay, and any adverse events and/or local irritation at injections site will be registered.
5. When all recordings have been finished, subjects are allowed to leave the site, but not before 8 hours after drug administration.
6. Drug accountability will be performed via **IVRS**.

<u>Day 2 and Day 3</u>

Subjects will return to site the day after drug administration as well as Day 3. The following data and samples will be recorded and collected:

1. Vital signs
2. Haematology
3. Biochemistry
4. Urinalysis
5. Hormones
6. Sampling for molecular biomarkers
7. Sampling for serum/plasma biomarkers
8. Concomitant medication

9. Local tolerability
10. Adverse events and toxicities
11. PK sample

PGA, BSA, PASI and VAS (if applicable) will only be assessed at Day 2.

Day 15, Day 29 and Day 43 - dose administrations

Subjects will come for drug administration at Day 15, day 29 and day 43. **Prior to trial product administration** the following data and samples should be recorded and collected:

1. Vital signs
2. Body measurements (weight only)
3. ECG
4. Blood pressure
5. Haematology
6. Biochemistry
7. Urinalysis
8. Hormones
9. BSA
10. PGA
11. PASI and VAS (if applicable)
12. Sampling for serum/plasma biomarkers
13. Concomitant medication
14. Local tolerability
15. Adverse events
16. PK sample (through value)
17. IVRS dispensing call

Thereafter trial product administration will occur.

Samplings and recordings occurring after trial product administration:

1. ECG will be recorded at 2 and 4 hours after each drug administration.
2. Blood pressure will be measured at 2 and 4 hours after each drug administration.
3. After each drug administration subjects will be closely monitored during their 4-hour-stay.
4. Drug accountability will be performed via **IVRS.**

Day 15: Sampling for molecular biomarkers, lesional skin biopsies (1x6 mm to be split in half). The skin biopsy procedure is optional.

Day 43: Sampling for antibodies.

Subjects are allowed to leave the site 4 hours after drug administration when all samples have been collected and all assessments have been recorded.

Day 44

Subjects must return to site for a 24-hour PK sample after the last drug administration.

Day 57 to Day 148 (± 3 days)

Subjects will come for regular visits at Day 57, 71, 99, 127 and 148. From Day 57 and onwards a deviation of ± 3 days from the scheduled day will be allowed. The following data and samples will be recorded and collected at each of those visits:

1. Vital signs
2. Haematology
3. Biochemistry
4. Urinalysis
5. Hormones
6. Sampling for serum/plasma biomarkers
7. PK samples
8. Concomitant medication
9. Local tolerability
10. Adverse events

Procedures which are not performed at every visit are listed below together with the time point at which they should be performed:

Day 57 (± 3 days): Lipids, ECG, BSA, PGA, PASI and VAS (if applicable), and quality of life questionnaire. At trial Day 57 all subjects must have been **fasting since midnight the same day** (only water will be allowed from midnight until collection of blood samples).

Day 99 (± 3 days): BSA, PGA, PASI and VAS (if applicable), and sampling for antibodies. Collection of lesional skin biopsies (1x6 mm to be split in half) is optional.

Day 148 (± 3 days): Body measurements (weight only), physical examination, ECG, BSA, PGA, PASI and VAS (if applicable), sampling for antibodies, and quality of life questionnaire.

8.1.4 Recordings

Exact dosing times, exact sampling time points for PK samples, and other serum, blood or plasma samples will be recorded directly in the electronic case record form (eCRF). Furthermore, PASI, PGA, BSA and VAS (if applicable) scores will be assessed/calculated and recorded in the eCRF.

Screening failures will be defined as those subjects who do not fulfil the eligibility criteria. These subjects are not eligible for the trial. At a minimum the reason for exclusion must be recorded in the screening failure form. Retesting of hepatic function (ALT and AST) is permitted within one week if first test is elevated. Screening visits are permitted to be rescheduled within one week, without taking new samples.

The screening failure form will be entered into the eCRF.

An overall interpretation of the ECG will be recorded in the eCRF and the recordings will be kept at site. Laboratory results will be entered in the Oracle Clinic data base.

Subjects may be identified through IVRS in the MD dose-escalation phase and MD expansion phase.

Subjects enrolled in the trial should be provided with a card stating that he/she is in a trial, contact address and telephone number.

In case a subject is being prematurely withdrawn from the trial the Investigator will ensure that the procedures for the last visit is undertaken, if possible. The primary reason (adverse event, non-compliance with protocol or other) for discontinuation must be specified in the eCRF. Even if the subject is not able to attend, the end of trial form must be completed and drug accountability must be performed.

8.1.5 Subject related information and assessments

Demography: Information about date of birth (age), sex and ethnicity will be recorded.

Medical history: Subjects will be asked about previous diseases, specifically about malignancies, active herpes/herpes zoster/cold sores, tuberculosis, lymphoproliferative diseases, hepatitis, HIV, and infectious diseases requiring systemic treatment within the last 2 weeks, as well as squamous or basal cell carcinoma within the last year.

Concomitant illnesses: From trial Day 1 and onwards subjects will be asked about onset of new diseases, developing during the course of the trial. Specific tests to determine if the subject is infected by hepatitis B and C and/or tuberculosis will be performed during screening only.

Test for hepatitis B and C: A blood test to determine if the subject is positive for hepatitis B and/or C will be taken at screening.

Test for tuberculosis: A test to determine if the subject is positive for tuberculosis will be taken at screening.

Body measurements: The height and weight of the subject will be recorded and the body mass index (BMI) will be calculated as:

$$BMI = weight\ (kg)/[\ height\ (m)\ ]^2$$

Pregnancy test (if applicable): Only women of non-childbearing potential and postmenopausal women (for at least 1 year) will be included. Otherwise FSH levels must be at least 40 mIU/mL, but if in doubt a regular urine pregnancy test will be performed.

8.2 Assessments for safety and tolerability

Safety will be regularly evaluated during the trial. This is done by recording adverse events and toxicity to Anti-IL-20 (109-0012) 100 mg/vial. Adverse event severity is defined by NCI's CTCAE, version 3. [23]

8.2.1 Adverse events

Adverse events, including local irritation at injection site, will be checked regularly during the entire trial.

Local irritation at injection site:
If an injection site reaction is observed, it must be recorded as an adverse event, with a specific description of the type of event such as:

- Redness
- Oedema
- Itching
- Burning
- Numbness
- Pain
- Eczema

The time of onset, duration and the size of the area must also be reported.

The following parameters are regarded as secondary safety endpoints and will be evaluated throughout the trial:

- Vital signs (blood pressure, pulse, and body temperature)
- 12-lead ECG
- Laboratory safety parameters haematology, biochemistry, hormones lipids, and urinalysis
- Immunogenicity
- Physical examination

8.2.2 Vital signs

Supine systolic and diastolic blood pressure as well as pulse should be measured after 5 minutes rest in supine position. Tympanic temperature should also be measured.

8.2.3 12-lead ECG

A standard 12-lead ECG will be performed while the subjects are in supine position. The evaluation should be recorded in the eCRF and judged as one of the following at screening visit:

- Normal
- Abnormal, not clinically significant
- Abnormal, clinically significant

ECG evaluations will be performed centrally.

8.2.4 Laboratory safety parameters

Biochemistry:

Adiponectin
Alkaline phosphatase (ALP)
Aspartate aminotransferase (AST)
Alanine aminotransferase (ALT)
Albumin
Calcium
Calcitonin
Chloride
CRP (C-reactive protein)
Creatinine
Creatinine kinase
Gamma glutamyltransferase
Glucose
Homocystein

Lactate dehydrogenase
Magnesium
Phosphate
Potassium
Sodium
Total bilirubin
Total protein
Urea
Uric acid

Haematology:
Basophils
Eosinophils
Erythrocytes
Erythrocyte sedimentation rate (ESR)
Haemoglobin
Haematocrit
Leucocytes
Lymphocytes (differential counts)
Monocytes
Neutrophils
Total erythrocyte and leukocyte count
Thrombocytes

Urinalysis: pH, protein, glucose, blood, bilirubin.

Lipids (only for the MD dose-escalation and MD expansion phases): Cholesterol, low density lipoprotein (LDL) cholesterol, high density lipoprotein (HDL) cholesterol. Subjects must be fasting from midnight the same day of sample collection, only water is allowed.

Hormones: Thyroid stimulating hormone-P (TSH), IL-1β, IL-6, TNF-α , INF-γ and follicle stimulating hormone (FSH) if applicable.

8.2.4.1 Handling of blood and urine samples

The analyses of haematology, biochemistry, hormones, lipids and urine samples will be performed at the local laboratory for the SD dose-escalation phase and at the central laboratory for the MD phases, according to their standard methods. The handling procedures of these samples will be performed according to the laboratory manual. Values outside standard ranges will be noted and the

Investigator must evaluate if the results are likely to be clinically significant or not. Clinically significant laboratory values qualify as adverse events and must be reported (see Section 12.2). Laboratory results must be signed electronically by the Investigator within 24 hours after receipt. The analytical reports must be sent to Novo Nordisk.

8.2.5 Immunogenicity

Immunogenicity (antibodies to 109-0012) samples will be analysed at Novo Nordisk using assays validated to detect the presence of antibodies against 109-0012 in human serum. Analysis for antibodies during the SD dose-escalation phase will only be performed if there are any safety concerns which are believed to be correlated to antibody production.

8.2.5.1 Handling of immunogenicity samples

After blood sampling (in normal serum sampling tubes) samples should be kept at room temperature for 1 hour before centrifugation to generate at least 0.5 mL serum, where possible. Serum should be transferred to cryotubes and stored at -20 °C. The tubes should be labelled with:

- Study ID
- Subject ID
- Nominal sampling time point
- Date of sampling
- Visit No.

The samples should be stored and shipped at -20 to -35°C.

8.2.6 Physical examination

Will be performed at the first and last visit and will include:

- Head, eyes, ears, nose, throat
- Respiratory system
- Cardiovascular system
- Gastrointestinal system
- Musculoskeletal system
- Central and peripheral nervous system
- Skin
- Lymph node palpation
- Genito-urinary system including breasts
- General appearance

8.3 Assessments for efficacy

Efficacy is a secondary endpoint for the SD and MD dose-escalation phases. In the MD expansion cohort, efficacy will be a primary endpoint, evaluated by PASI, PGA and VAS.

8.3.1 Body surface area (BSA)

The body area affected by psoriasis is estimated as a function of the severity of the disease. BSA is easily evaluated by the number of subject's hand areas affected. The area of one side of the flat hand of the subject is estimated to account for 1% of the total BSA, but this generally results in an overestimation. BSA is therefore not optimal for defining psoriasis severity, also because there are subjects with low BSA involvement who have very severe psoriasis and some subjects with high BSA involvement who have mild psoriasis. The BSA assessment is therefore recommended to be supplemented by other assessments.[24]

8.3.2 Psoriasis Area and Severity Index (PASI)

The PASI score is generated by dividing the body into four main areas (head, arms, trunk to groin, and legs to top of buttock) for which an average score of scaling, erythema and thickness is assessed (0-4, with 4 being most severe). The scores for the four different areas are added together to obtain an overall severity rating. This sum should be multiplied with the figure used for converting the percentage of the skin area covered with psoriasis for each of the four body areas (i.e. 0= 0% of body area, 1= < 10%, 2= 10-< 30%, 3= 30-<50%, 4= 50-<70%, 5= 70-<90%, 6= 90-100%). The four figures obtained should then be multiplied by 0.1, 0.2, 0.3 and 0.4 for head, arms, trunk and legs, respectively. The sum of these four values is the final PASI score. [25, 26] See formula below:

Head: $0.1(R+T+S)E = V$

Arms: $0.2\,(R+T+S)E = X$

Trunk: $0.3\,(R+T+S)E = Y$

Legs: $0.4\,(R+T+S)E = Z$

where: R = score for redness (0-4)

T = score for thickness (0-4)

S = score for scaliness (0-4)

E = score for extent (0-6)

The sum of V+X+Y+Z gives the total PASI score which can range from 0 to 72.

8.3.3 Physicians Global Assessment (PGA) score

The PGA is a six-point score that summarizes the overall quality (erythema, scaling and thickness) and extent (BSA) of plaques where 1 being clear, 2 minimal, 3 mild, 4 moderate, 5 severe, and 6 being very severe psoriasis.[25] Please see below:

Table 8–1 PGA score definition

Clear (1)	Plaque thickening = none (no elevation or thickening over normal skin)<br>Scaling = none (no evidence of scaling)<br>Erythema = none or slight (hyperpigmentation or residual red colouration)
Minimal (2)	Plaque thickening = none or slight (possible but difficult to ascertain whether there is a slight elevation above normal skin level)<br>Scaling = none or slight (residual surface of dryness and scaling)<br>Erythema = up to mild (up to light red or pink colouration)
Mild (3)	Plaque thickening = slight (slight but definite elevation)<br>Scaling = fine (fine scales partially or mostly covering lesions)<br>Erythema = up to moderate (up to definite red colouration)
Moderate (4)	Plaque thickening = moderate (moderate elevation with rounded or sloped edges)<br>Scaling = coarser (most lesions at least partially covered)<br>Erythema = moderate (definite red colouration)
Severe (5)	Plaque thickening = marked (moderate elevation with rounded or sloped edges)<br>Scaling = coarser (most lesions at least partially covered)<br>Erythema = moderate (definite red colouration)
Very severe (6)	Plaque thickening = very marked (very marked elevation typically with hard or sharp edges)<br>Scaling = very coarse (thick tenacious scale covers most or all of the lesions)<br>Erythema = very severe (extreme red colouration, deep red colouration)

8.3.4 Visual Analogue Scale (VAS)

The VAS is a self-rating scale where subjects with psoriatic arthritis judge their current disease state by marking the scale, from no pain at the left end (0 cm) to very severe pain at the right (10 cm) on a piece of paper, which will be included in the patient´s record.

8.3.5 Quality of life, DLQI

The 10 items of the Dermatology Life Quality Index (DLQI) can be used to calculate scores for the following six subscales: symptoms and feelings, daily activities, leisure, work and school, personal relationships, and treatment satisfaction. A DLQI total score is the sum of the 10 equal-weighted items and ranges from 0 to 30, with higher scores indicating poorer quality of life. DLQI has been used quite extensively during the last few years in the assessment of the new biologic treatments for psoriasis.

8.4 Other assessments

In total, approximately 250 mL blood will be collected during the SD dose-escalation phase and 370 mL for the MD dose-escalation and expansion phases, respectively. For each visit, regardless of trial phase, approximately 30 mL of urine will be collected. In addition, skin biopsies will be collected (not mandatory), but only during the MD dose-escalation phase and the MD expansion phase. Irrespective of site for analysis, all samples should be sent to the local laboratory (SD dose-escalation phase) or central laboratory (MD phases), which then will either store them, transfer them or analyse them. For more specific information, please refer to the text below and to table Table 8–2.

Table 8–2 Samples and assays

Assays	Place of assay	Specification
**Pharmacodynamic Biomarkers (serum/plasma)**		
• IL-20	• Novo Nordisk A/S, Denmark	• 5 mL blood in total. The serum is to be divided into a number of aliquots and stored for analysis of IL-20 and other serum markers.
• sCD25	• Central/local laboratory, USA	• See above
• Other serum markers	• NN referral laboratory	• See above
• Plasma markers	• NN referral laboratory	• 5 mL blood in total. The plasma is to be divided into a number of aliquots and stored for assessment of plasma markers.
**Pharmacodynamic Biomarkers (tissue)**		
• Histopathology and immunohistochemistry	• NN referral laboratory	• Non-lesional and lesional skin biopsies (cryo preserved), for MD dose-escalation and MD expansion phase only
**Pharmacogenomic Biomarkers (blood)**		
• Genotyping	• NN referral laboratory	• Blood drops on Whatman FTA cards (dry and ambient storage), MD expansion phase only
• Gene expression	• Novo Nordisk A/S, Denmark	• 2.5 mL blood in PAXgene tubes
**Pharmacogenomic Biomarkers (tissue)**		
• Gene expression	• Novo Nordisk A/S, Denmark	• Lesional skin biopsies (cryo preserved), for MD dose-escalation and MD expansion phase only
**Pharmacokinetics**	Novo Nordisk A/S, Denmark	2.5 mL serum
**Immunogenicity**	Novo Nordisk A/S, Denmark	0.5 mL serum
**Haematology**	Central/local laboratory, USA	7 mL blood
**Biochemistry (incl. lipids and hormones)**	Central/local laboratory, USA	10 mL serum

8.4.1 Pharmacokinetic samples

Pharmacokinetic endpoints to be analysed: C_{max}, t_{max}, terminal $t_{1/2}$, R_{acc}, $AUC_{0-\tau}$, AUC_{0-t} and AUC, if possible.

PK samples will be collected regularly up to 24 hours after dose administration and thereafter at each of the following 7 visits, in the SD dose-escalation phase.

During the MD dose-escalation phase and MD expansion phase, PK samples will be collected before dosing (0 hours) at each dosing visit and at 1, 2, 4 and 24 hours after dosing for the first and last dose. PK samples will also be collected at all other visits during the trial. Actual sampling time must be recorded in the eCRF.

After each dose level, the bioanalysis results will be sent electronically to the representative in DMPK & Bioanalysis, Novo Nordisk A/S, for interim PK calculations. The preliminary calculation of the PK parameters will be performed after each dose level, and will be based on the nominal time points for the dose administration and PK sampling. The serum PK parameters for 109-0012 will be calculated by non-compartmental methods according to current guidelines. The preliminary pharmacokinetic results will be available at the safety evaluation meetings and will support the dose-escalation decisions during the clinical phase of the trial.

The final PK analysis will be performed by Biostatistics, Novo Nordisk A/S by non-compartmental methods. The final pharmacokinetic analysis will take place following completion of the clinical phase of the trial, and will be based on audited data of the serum concentrations of 109-0012, as well as final Quality Controlled time points as recorded in the database. The determination of terminal rate constant values will be based on input from DMPK & Bioanalysis, Novo Nordisk as well as decisions taken at the database release meeting. The final pharmacokinetic results will be included in the Clinical Trial Report.

8.4.1.1 Sampling and handling (PK samples)

Serum will be analysed by use of an enzyme-linked immunosorbent assay (ELISA) specific for 109-0012. The ELISA is validated for human serum samples. Results will be reported initially as stated in 8.4.1. Final results will be reported in a bioanalytical report.

8.4.1.2 Blood Sampling Procedure and Sample Handling (PK samples)

The blood sample will be centrifuged and the separated serum will be transferred into labelled tubes. The serum samples will be stored frozen (-15° C or below) pending shipment to central laboratory. The central laboratory will immediately transfer samples to DMPK&BA, Novo Nordisk A/S. All shipments will be on dry ice.

8.4.1.3 Serum Sample Labelling (PK samples)

The tubes will be labelled by the central laboratory and must include the following information:

- Trial ID
- Visit No.
- Subject ID
- Nominal time

8.4.1.4 Shipment of Samples (PK samples)

The samples for pharmacokinetic assessment will be transported frozen at -15°C or below, in an appropriate packaging material containing dry ice. A list identifying all planned samples, noting missed samples or breakage of samples, will be enclosed.

A copy of the treatment report will be forwarded to the persons responsible for the PK drug analysis of 109-0012 in DMPK&BA, in order to facilitate the drug analysis work of the PK samples. Only the predose and expected C_{max} sample from placebo subjects will be analysed.

8.4.2 Pharmacodynamic Biomarker Assessments

Serum and plasma samples

In psoriasis, several pro-inflammatory mediators have been investigated as markers of early disease control.[28] In addition, the level of sCD25 has previously been shown to correlate to the PASI score during treatment.[7] In order to search for pharmacodynamic biomarkers of systemic 109-0012 effects and other biomarkers related to psoriasis and co-morbidities, the assessments may include but will not be limited to:

- Levels of serum IL-20 and sCD25
- Proteomic profiling of serum and plasma biomarkers, e.g. by multi-analyte profiling

Results from proteomic profiling will be analysed at a later time point and reported separately and appended to the clinical trial report.

Skin biopsies

Hyper proliferation of keratinocytes and epidermal infiltration with both T cells and dendritic cells are hallmarks of psoriasis and $CD68^+/CD11^+$ myeloid cells have been suggested to be an important source of IL-20.[29] In addition to keratinocytosis and leukocyte infiltration, increased angiogenesis is an early prominent event in psoriasis.[30] In order to search for morphological markers of 109-0012 effects, skin biopsies will be collected and subjected to histopathological and immunohistochemical analyses. Lesional biopsies (1x6 mm), obtained from the centre of the lesion, will be collected at baseline and at trial Week 3 and trial Week 15. Each biopsy will be split into two equal halves (1x3 mm) and subjected to cryo preservation. Furthermore, a non-lesional biopsy (1x3 mm, cryo

preservation) will be collected, but only at baseline. The assessments (for the non-lesional and one of the half lesional biopsies) may include but will not be limited to:

- Histopathological scoring including assessment of epidermal thickness and proliferation, e.g. by H&E, keratin 16, and Ki67 stain
- Exploratory assessment of 109-0012 effects on skin, vasculature, and immune system:
  - expression of IL-20 and IL-20 receptors
  - infiltrating leukocyte subsets, e.g. CD3, CD11c, and CD68
  - IL-20 signalling, e.g. pSTAT3
  - angiogenesis, e.g. CD31 stain

Results from skin biopsy analyses will be analysed at a later time point and reported separately and appended to the clinical trial report.

8.4.3 Pharmacogenomic Biomarker Assessments

Pharmacogenomic biomarkers assessments will only be performed for subjects who have given specific consent to this part of the trial.

Recently, microarray analysis of *ex vivo* IL-20 stimulated reconstituted human epidermis and keratinocytes have indicated a role for IL-20 subfamily cytokines as important regulators of epidermal keratinocyte biology with potentially pivotal roles in the immunopathology of psoriasis.[31, 32] In support, IL-20 gene polymorphisms have previously been linked to plaque-type psoriasis predisposition.[33] In search for molecular biomarkers related to 109-0012 safety, efficacy, MoA as well as to the pharmacodynamic end-points gene expression patterns and genotyping will be analysed in whole blood samples. The assessments may include but will not be limited to:

Blood samples

- Gene expression analysis by gloal transcriptome profiling and qRT-PCR of selected genes related to systemic 109-0012 effects
- Genotyping of IL-20 SNP haplotypes

Skin biopsies

Lesional biopsies (1x6 mm), obtained from the centre of the lesion, will be collected at baseline and at trial Week 3 and trial Week 15. Each biopsy will be split into two equal halves (1x3 mm) and subjected to cryo preservation. One of the halves (1x3 mm) will be analysed for:

- Gene expression analysis by global transcriptome profiling and qRT-PCR of selected markers related to psoriasis and 109-0012 effects on skin, vasculature, and immune system

Results from gene expression analyses will be analysed at a later time point and reported separately and appended to the clinical trial report.

8.4.4 Reporting of pharmacodynamic and pharmacogenomic biomarker assessments

The pharmacodynamic biomarker analyses and the pharmacogenomic analyses may include genes that are unknown or have not been included in the scientific hypotheses at the present time of trial, but that, during the collection of data from this trial, may evolve as new candidate genes and markers related to 109-0012 safety, efficacy, or mechanism of action. Any analyses that are not planned at the present point in time will only be related to and used in connection with the data collected in the present trial, and the subject's identity will remain confidential. Biopsies, aliquots of serum and plasma and blood samples will be stored for up to 15 years after completion of the trial where after all samples will be destroyed. The analyses will not have any medical consequences for the subject or their relatives.

8.4.5 Sampling and handling of blood, serum, plasma and tissue samples

8.4.5.1 Serum samples for biomarker analyses

At the trial site, 5 mL blood samples will be centrifuged in plain tubes and the separated serum will be transferred into a number of 0.25 mL aliquots of labelled tubes. The serum samples will be stored frozen (-80°C or below) until shipment to the local/central laboratory. At the laboratory, samples will be stored frozen (-80°C or below) until further transfer. All shipments will be on dry ice.

8.4.5.2 Labelling of Serum Samples

The label must include the following information:

- Serum biomarkers
- Trial ID
- Visit No.
- Subject ID

Aliquots of serum will be analysed for levels of IL-20 and sCD25 by use of enzyme-linked immunosorbent assay (ELISA) assays validated for human serum samples:

- Levels of IL-20 will be analysed at Novo Nordisk A/S.
- Levels of sCD25 will be analysed at the central laboratory

8.4.5.3 Plasma Samples for Biomarker Analyses

At the trial site, 5 mL blood samples will be centrifuged in EDTA tubes and the separated plasma will be transferred into a number of 0.25 mL aliquots of labelled EDTA tubes. The plasma samples will be stored frozen (-80°C or below) until shipment to the local/central laboratory. At the laboratory, samples will be stored frozen (-80°C or below) until transfer. All shipments will be on dry ice.

Labelling of serum samples will include the following information:

- Plasma biomarkers
- Trial ID
- Visit No.
- Subject ID

8.4.5.4 Blood Samples for Gene Expression Analyses

At the trial site, 2.5 mL blood samples will be collected into PAXgene tubes according the manufacturers instructions. Samples will be stored frozen (-80°C or below) until shipment to the local/central laboratory. At the laboratory, samples will be stored frozen (-80°C or below) until transfer. All shipments will be on dry ice.

Labelling of samples will include the following information:

- Blood gene expression
- Trial ID
- Visit No.
- Subject ID

Samples will be analysed at Novo Nordisk A/S.

8.4.5.5 Blood Samples for Genotyping

At the trial site, blood drops will be collected on Whatman FTA cards according the manufacturers instructions. Samples will be stored dry and ambient until shipment to the local/central laboratory. At the laboratory, samples will be stored dry and ambient until shipment. All shipments will be ambient.

Labelling of samples will include the following information:

- Genotyping
- Trial ID
- Subject ID

Samples will be analysed at NN referral laboratory.

8.4.5.6 Tissue Samples for Biomarker Analyses

At the trial site, 1x6 mm lesional biopsies will be collected and split into two equal halves immediately upon sampling at baseline, trial Week 3 and 15. Both parts will be cryopreserved for immunohistochemical, histopathological and gene expression analyses. A non-lesional biopsy (1x3

mm) will also be collected, at baseline only, and cryo preserved for immunohistochemical and histopathological assessments.

The samples will be transferred to screw cap cryotubes and stored frozen (-80°C or below) until shipment to the central laboratory. At the local/central laboratory, samples will be stored frozen (-80°C or below) until shipment. All shipments will be on dry ice.

Labelling of tubes for tissue samples will include the following information:

- Sample type, i.e. “Tissue histo L”, “Tissue histo NL”, “Tissue immuno L”, “Tissue immuno NL”, or “Tissue gene expression”
- Trial ID
- Visit No.
- Subject ID

Tissue samples will be subjected to histopathological, immunohistochemical, and microarray analyses, respectively:

- Histopathological assessments will be conducted at NN referral laboratory
- Immunohistochemical assessments will be conducted at NN referral laboratory
- Microarray analyses will be conducted at Novo Nordisk A/S

8.4.6 PK/PD modelling

In addition to the non-compartmental (NCA) PK evaluation, a population PK/PD model will be developed (see 17.5 for more details).

8.5 Subject compliance

The trial products (active drug or placebo) will be injected subcutaneously in the abdomen by qualified site personnel, who must be trial independent in order to keep the blinding. Furthermore, within an hour before administration, a trial independent pharmacist or corresponding must prepare the syringes with either placebo or active trial product.

9 Trial supplies

9.1 Trial products

The following trial products will be supplied by Novo Nordisk A/S:

- Anti-IL-20 (109-0012) 100 mg/vial as sterile freeze-dried powder in single use vials containing 127.5 mg drug substance. One 9 mL powder-containing vial is to be reconstituted with 1.1 mL of sterile Water for Injection, in order to obtain a concentration of 100 mg/mL.
- Placebo solution (Placebo (109-0012) 0 mg/mL) in single use vials containing 9 mL.
- Diluent solution (Placebo (109-0012) 0 mg/mL) in single use vials containing 9 mL to be used for preparation of single use vials of 10 mg/mL and 1 mg/mL strength solutions of 109-0012 for the dose-escalation phases.

The reconstituted Anti-IL-20 (109-0012) 100 mg/vial, as well as the Placebo (109-0012) 0 mg/mL, are colourless/almost clear solutions with pH of 6.5. It is recommended to use the trial drug immediately following reconstitution. The reconstituted preparations should be transferred to the dosing syringe no earlier than 1 hour prior to trial drug administration and stored below 25 °C (no freezing).

Detailed instructions regarding reconstitutions and dilution will be given in the Trial Materials Manual (TMM).

Appropriate volumes of Anti-IL-20 (109-0012) 100 mg/vial or Placebo (109-0012) 0 mg/mL will be administered, based on weight, as subcutaneous injections into the abdomen using a proper syringe. Please see the TMM for details.

The trial products are produced according to Good Manufacturing Practice. A TMM, detailing instructions on storage, handling and administration of the trial products, will be provided by Novo Nordisk. No trial product may be dispensed to any person except to the subjects enrolled into the trial.

9.2 Packaging and labelling of trial products

Novo Nordisk A/S will label and pack the trial products.

Trial product will be packed in non subject specific boxes with a unique Dispensing Unit Number (DUN), the DUN of each individual vial contained in a box is identical to the box DUN. The IVRS

will allocate the DUN number(s) to be used for each subject in the MD dose-escalation and MD expansion phase.

Labelling will be in accordance with local law and trial requirements.

9.3 Storage and drug accountability of trial products

The trial products must be stored in a secure place under refrigeration at 2-8°C until use and is stable until the expiry date. The investigator must ensure the availability of proper storage conditions and record and evaluate the temperature. Calibration of the temperature monitoring unit must be done at least once a year. Preferably the temperature must be recorded by continuous, automatic, stationary equipment. It should be placed in the immediate vicinity of trial product. If the equipment does not record the temperature continuously the temperature must be logged at least once every working day. The equipment must have a "memory function" of temperatures (e.g. min/max temperatures are recorded) for holidays and the temperature must be logged the next working day.

In case of incorrect storage the monitor must be contacted without delay and the trial product must be set on-hold and not dispensed to subjects until notified by the monitor.

No trial product may be dispensed to any person except to the subjects enrolled into the trial.

Unused trial product must be stored separately from used trial product.

All trial products must be retained for inspection by the monitor after completion of the trial. The monitor will, upon completion of drug accountability, arrange for destruction of used, unused and broken vials of the supplied trial product.

9.4 Auxiliary supply

All auxiliary supplies will be dispensed from the site facility.

10 Randomisation, breaking of blinded codes and interactive voice response system (IVRS)

10.1 Randomisation and blinding

The trial will be conducted as a randomised double-blinded trial, with a SD dose-escalation phase and a MD dose-escalation phase running in semi-parallel. After completion of the SD and MD dose-escalation phases, an MD expansion phase, at the maximum tolerated dose level, will be initiated. A MD dose cohort at a higher dose level than the SD dose cohort can not be initiated before the dose cohort in the SD phase has been completed.

Each clinical site will be supplied with boxes containing trial product. Each box of trial product will be labelled with a unique Dispensing Unit Number (DUN), the DUN of each individual vial contained in a box is identical to the box DUN. A subject can thus be allocated to more than one DUN per cohort he/she participates in.

At screening the subjects will be assigned the lowest available subject number, which will be used throughout the trial.

IVRS will allocate DUNs for the MD dose-escalation phase and the MD expansion phase.

In order to keep the trial site staff blinded, an unblinded, and otherwise trial-independent coordinator/pharmacist must be involved at each site in order to prepare the trial products and fill the syringes. Furthermore, a trial-independent nurse/site personnel must administer the trial product.

If subjects drop out before completion of trial Day 29 in the SD dose-escalation phase, and trial Day 57 in the MD phases, they must be replaced. Replacement should always occur to the same dose cohort as to which the subject who dropped out belonged. Randomisation lists will be provided by Clinical Supply Coordination.

10.2 Breaking of blinded codes

The code for a particular subject may be broken in a medical emergency, if knowing the identity of the treatment allocation would influence the treatment of the subject. Whenever a code is broken, the person breaking the code must record the time, date and reason as well as his/her initials in the source documents. The subject should be withdrawn from the trial if the code is broken.

If the trial site needs to break the code, the sponsor should, if possible, be contacted prior to breaking the code. In all cases, the monitor must be notified within 24 hours after the code has been broken.

The treatment will be accessible for Investigator, Novo Nordisk Affiliate and International Product Safety, using IVRS for code breaking. The contingency plan for code breaking is using ClinPhone Support, available 24/7.

The central laboratory will not be provided with treatment reports. However, a copy of the treatment reports will be forwarded to the person responsible for bioanalysis of 109-0012 in DMPK & Bioanalysis, Novo Nordisk A/S, in order to facilitate the availability of intermediate PK results to be used for the safety evaluation during the trial. A copy of the the treatment reports will also be forwarded to the person responsible for PK/PD analysis in Biomodelling, Novo Nordisk A/S, in order to begin exploratory model development to assess the pharmacokinetic/pharmacodynamic properties of 109-0012.

10.3 Interactive voice response system (IVRS)

For information about when to use IVRS, please see section 8.

11 Concomitant illnesses and medication

Definitions:

Concomitant illness: any illness that is present at the start of the trial (i.e. at the first visit).

Concomitant medication: any medication other than the trial products that is taken during the trial, including the screening and run-in periods.

Details of all concomitant illnesses and medication must be recorded at trial entry (i.e. at the first visit). Any changes in concomitant medication must be recorded at each visit. If the change influences the subject's eligibility to continue in the trial, the sponsor must be informed.

The information collected for each concomitant medication includes, at a minimum, start date, stop date or continuing and indication.

12 Adverse events

12.1 Definitions

Adverse event (AE):

An AE is any undesirable medical event occurring to a subject in a clinical trial, whether or not related to the trial products.

Note: This includes events from the first trial related activity after the subject has signed the informed consent and until post-treatment follow-up period as defined in the protocol.

The following should not be recorded as AEs:

- Pre-planned procedure unless the condition for which the procedure was planned has worsened from the first trial related activity after the subject has signed the informed consent.
- Pre-existing conditions found as a result of screening procedures. These should be recorded as medical history/concomitant illness.

An AE can also be a clinical laboratory abnormality regarded as clinically significant i.e. an abnormality that suggests a disease and/or organ toxicity and is of a severity which requires active management (i.e. change of dose, discontinuation of trial product, more frequent follow-up or diagnostic investigation).

Serious adverse event (SAE):

An SAE is an experience that at any dose results in any of the following:

- Death
- A life-threatening* experience
- In-patient hospitalisation** or prolongation of existing hospitalisation
- A persistent or significant disability/incapacity
- A congenital anomaly/birth defect
- Important medical events that may not result in death, be life-threatening*, or require hospitalisation** may be considered an SAE when, based upon appropriate medical judgement, they may jeopardise the subject and may require medical or surgical intervention to prevent one of the outcomes listed in this definition

* The term life-threatening in the definition of SAE refers to an event in which the subject was at risk of death at the time of the event. It does not refer to an event which hypothetically might have caused death if it was more severe.

* * The term hospitalisation describes a period of at least 24 hours. Over-night stay for observation, treatment at emergency room or treatment on an out-patient-basis does not constitute a hospitalisation. However, medical judgement must always be exercised, and when in doubt, the case should be considered serious. Hospitalisations for administrative, trial related and social purposes do not constitute hospitalisations as defined by the seriousness criteria for SAEs and should therefore not be reported as such. Likewise, hospital admissions for surgical procedures planned prior to trial inclusion are not considered adverse events.

Non-serious adverse event:

A non-serious AE is any AE which does not fulfil the definition of a serious AE.

Relationship to trial product* assessment definitions:

The relationship of an adverse event should be assessed using the following categories:

- Probable: good reasons and sufficient documentation to assume a causal relationship
- Possible: a causal relationship is conceivable and cannot be dismissed
- Unlikely: the event is most likely related to an aetiology other than the trial product

* Trial product is defined as Anti-IL-20 (109-0012) 100 mg/vial formulation or the corresponding placebo formulation.

Outcome categories and definitions:

- Recovered: fully recovered or by medical or surgical treatment the condition has returned to the level observed at the first trial related activity after the subject signed the informed consent
- Recovering: the condition is improving and the subject is expected to recover from the event. This term should only be used when the subject has completed the trial
- Recovered with sequelae: as a result of the AE, the subject suffered persistent and significant disability/incapacity (e.g. became blind, deaf, paralysed). Any AE recovered with sequelae should be rated as an SAE
- Not recovered
- Fatal
- Unknown: this term should only be used in cases where the subject is lost to follow up.

Coding:

All adverse events will be coded in MedDRA, current version.

Severity assessment

The National Cancer Institute's (NCI) Common Terminology Criteria for Adverse Events (CTCAE) Version 3.0 will be used to assess and grade severity for adverse events and for laboratory abnormalities judged to be clinically significant.

The CTCAE Version 3.0 is a descriptive terminology with a grading (severity) scale provided for each AE term. Within each category, AEs are listed accompanied by their descriptions of severity (Grade 1-5). Each AE term will be mapped to a MedDRA term and code. For this trial the CTCAE will only be used for grading the severity of an AE and not for causality assessment.

If the event is not covered in CTCAE Version 3.0, the guidelines shown in Table 12–1 should be used to assess severity.

Table 12–1 Severity and toxicity grade of events not covered by CTCAE

**Toxicity Grade/ Severity**		**Description**
Grade 1	Mild	Transient or mild discomfort; no limitation in activity; no medical intervention or therapy required. The subject may be aware of the sign or symptom but tolerates it reasonably well.
Grade 2	Moderate	Mild to moderate limitation in activity, no or minimal medical intervention/ therapy required.
Grade 3	Severe	Marked limitation in activity, medical intervention/ therapy required, hospitalisations possible.
Grade 4	Life-threatening	The subject is at risk of death due to the adverse event as it occurred. This does not refer to an event that hypothetically might have caused death if it was more severe.
Grade 5	Fatal	The subject died due to the event.

12.1.1 Defintion of dose limiting toxicities

Grade 3 AEs or higher will represent Dose Limiting Toxicities (DLT), except when the relationship to trial product is judged as unlikely.

12.1.2 Definition of maximum tolerated dose and maximum administered dose

The maximum tolerated dose (MTD) is the highest dose level below the maximum administered dose (MAD) where none or 1 subjects experience a DLT.

The maximum administered dose is the highest dose given in the trial.

12.2 Collection, recording and reporting of adverse events

All events meeting the definition of an AE must be collected and reported. At each contact with the trial site (visit or telephone, excluding safety visits, where the subject is not seeing the Investigator or his staff (e.g. visits to the laboratory)), the subject must be asked about adverse events e.g.: "Have you experienced any problems since the last contact?"

All AEs, either observed by the Investigator or reported by the subject, must be recorded by the Investigator and evaluated.

Sponsor's assessment of expectedness is done according to the reference documents: Novo Nordisk investigational product (Anti-IL-20 (109-0012) 100 mg/vial): IB, 1st Edition, and any updates hereof.

The Investigator should record the diagnosis, if available. If no diagnosis is available the Investigator should record each sign and symptom as individual adverse events.

All adverse events must be recorded by the Investigator on the standard adverse event form. If more than one sign or symptom is to be reported, use a separate adverse event form for each sign and symptom. For serious adverse events, the safety information form must also be completed.

The Investigator must report initial information on all serious AEs to Novo Nordisk within **24 hours** of obtaining knowledge about the event to:

Product Safety Surveillance Group. Fax: 609-987-5997

The Investigator must complete the adverse event form in the EDC application and the safety information form on the paper CRFs. The Investigator must forward electronically/fax/courier copies of the safety information form to Novo Nordisk within **5 calendar days** of obtaining knowledge about the serious adverse event.

The sponsor must inform the regulatory authorities and independent ethics committee (IEC)/institutional review boards (IRB) in accordance with the local requirements in force and International Conference on Harmonisation Good Clinical Practice (ICH GCP) [34].

The sponsor will notify the Investigator of trial product related suspected unexpected serious adverse reactions in accordance with the local requirements. In addition, the Investigator will be informed of any trial related procedure SAE which may warrant a change of any trial procedure.

Investigators will be notified of trial-related SAEs in accordance with the local requirements in force and ICH GCP [34].

The monitor must be informed accordingly.

12.2.1 Medical events of special interest

Events such as medication errors (e.g. wrong drug administration, overdose or wrong route of administration) and suspected transmission of an infectious agent via a trial product are always to be considered as medical events of special interest.

Medical events of special interest must always be reported to International Product Safety (IPS) on adverse event form and safety information form irrespective of seriousness within the same timelines as SAEs.

12.3 Follow-up of adverse events

During and following a subject's participation in a clinical trial, the Investigator/institution should ensure that adequate medical care is provided to the subject for any adverse events, including clinically significant laboratory values related to the trial. The Investigator/institution should inform the subject when medical care is needed for adverse events of which the Investigator becomes aware.

The follow up information should only include new (updated and/or additional) information that reflects the situation at the time of the Investigator's signature.

All non-serious AEs classified as severe or possibly/probably related to the trial product must be followed until the subject has recovered and all queries have been resolved. However, cases of chronic conditions can be closed with an outcome of "recovering" or "not recovered". **If subjects die from another event**, these cases can be closed with an outcome of "recovering" or "not recovered".

All other non-serious AEs must be followed until the outcome of the event is "recovering" (for chronic conditions), or "recovered" or until the end of the post-treatment follow-up stated in the protocol, whichever comes first, and until all queries related to these AEs have been resolved. **If subjects die from another event**, these cases can be closed with an outcome of "recovering" or "not recovered".

The Investigator must ensure that the worst case severity and seriousness is kept consistent. The Investigator must record follow-up information on non-serious adverse events by updating the adverse event form in the EDC application.

The Investigator must forward follow-up information on serious adverse events within 5 calendar days of obtaining the information. This must be done by updating the adverse event form in the EDC application and/or completing the safety information form on paper CRF and forwarding these to Novo Nordisk.

All serious adverse events must be followed until the outcome of the event is recovered, recovered with sequelae or fatal and until all queries have been resolved. For cases of chronic conditions and cancer or if the subject dies from another event follow-up until the outcome categories are "recovered", "recovered with sequelae" or "fatal" is not required, as these cases can be closed with an outcome of "recovering" or "not recovered".

After access to the EDC application is removed, the Investigator must record any SAE follow-up information, if required, on the paper CRFs provided at study closure.

12.4 Pregnancy

Women of child-bearing potential are not allowed to participate in the trial.

12.5 Precautions/over-dosage

Anti-IL-20 (109-0012) 100 mg/vial has not previously been used in humans and therefore no experience with overdose and overdose reactions exists. In case of an overdose of Anti-IL-20 (109-0012) 100 mg/vial, treatment should be suspended and symptomatic medical treatment according to the clinical condition should be applied. No antidote exists in case of overdose of Anti-IL-20 (109-0012) 100 mg/vial.

Any overdose should be reported as an AE, with or without clinical manifestations, see 12.2.1.

12.6 Safety committees

12.6.1 Internal Novo Nordisk safety committee

Novo Nordisk will establish an internal safety committee to perform ongoing safety surveillance. The safety committee may recommend unblinding of any data for further analysis. If so, an independent ad hoc group will be established to maintain the blinding, since unblinded subject data will be sent from IVRS.

A Study Safety Group will be established according to Novo Nordisk standard operating procedure with the responsibility to evaluate all safety data during the SD and MD dose-escalation phases, and to determine if progression to the next dose level can be supported. The Study Safety Group will also have the mandate to add another cohort other than for safety reasons, if relevant. The Study Safety Group will consist of:

- Head of Clinical Pharmacology (chair)
- Responsible Clinical Pharmacology Scientist/International Trial Manager
- Local Medical Director
- International Medical Officer

- Safety Surveillance Advisor from International Product Safety
- Representative from Department of DMPK & Bioanalysis

A summary report with safety and PK data will be distributed to members of the Safety Group and a meeting will be set up. The decisions taken at the meeting will be documented in minutes, and distributed to Safety Group members, investigators, Chairman for the Safety Committee, relevant Project Vice President and other relevant persons.

SD, dose-escalation phase

The safety summary report for the decision on dose-escalation in the SD dose-escalation phase will contain information on laboratory safety data, AEs including local tolerability, vital signs, ECG evaluations and subject related information for all subjects in the dose cohort through trial Day 15, as well as PK data through trial Day 15. In line with other safety laboratory analyses, acute phase cytokine response (IL-1β, IL-6, TNF-α , INF-γ) and homocystein and adiponectin response in the first 24 hour post dosing period must be available for the Safety Group meetings to be included as part of the overall safety evaluation prior to escalation to the next cohort.
PK data will be presented in such a manner that unblinding of the Safety Group is avoided.

MD, dose-escalation phase

The safety summary report for the decision on dose-escalation in the MD dose-escalation phase will contain information on laboratory safety data, AEs including local tolerability, vital signs, ECG evaluations and subject related information for all subjects in the dose cohort through trial Day 57, as well as PK data through trial Day 57. PK data will be presented in such a manner that unblinding of the Safety Group is avoided.

Sites must not proceed to the next dose level with the new dose cohort before the recommendation from the Novo Nordisk Study Safety Group to escalate has been issued. The Study Safety Group can also recommend to stop further dose-escalations, or to add another cohort (N=3+1) of patients to the last performed dose cohort based on safety (see Table 5–2) or due to inconclusive PK and/or clinical data. Dose cohorts in-between existing dose levels can also be added (see Table 5–2). Maximally 8 subjects can be treated at the same dose level.

13 Case report forms

Novo Nordisk will provide a system for electronic data capture (EDC). This system, and support services to the system, will be supplied by a clinical services vendor, Phase Forward. The activities of this vendor will be under the direction and supervision of Novo Nordisk.

13.1 Rules for completing eCRFs

Ensure that all relevant questions are answered and that no empty data blocks exist.

If a test/assessment has not been done and will not be available, or if the question is irrelevant (e.g. is not applicable) indicate this according to the data entry instructions.

The Investigator or Investigator's authorised staff must ensure that all information derived from source documentation is consistent with the source information. By signing the affirmation statement electronically, the Investigator confirms that the information is complete and correct.

13.2 Corrections to eCRFs

Corrections to the eCRF data will be made by the Investigator or the Investigator's authorised staff. An audit trail will be maintained in the EDC application containing as a minimum: Identification of the person entering the data, date and time of the entry and reason for the correction.

If corrections are made by the Investigator's authorised staff after the date of the Investigator's signature on the affirmation statement, the statement must be signed again by the Investigator.

13.3 eCRF flow

The Investigator must ensure that data is recorded in the eCRFs within 48 hours after the visit. When data is entered it will be available to Novo Nordisk for data verification activities.

Safety laboratory results will be electronically uploaded and must be electronically signed by the Investigator as soon as possible after they have been made available, or not later than 12 hours after being loaded.

Site specific eCRF data (in an electronic readable format) will be provided to the Investigator site after the trial database is released and access to update the trial data on the EDC application is removed. This data will be retained by the site.

When the final clinical trial report is available the data will be archived by Novo Nordisk.

14 Monitoring procedures

During the course of the trial, the Monitor will visit the trial site to ensure that the protocol has been adhered to, that all issues have been recorded, to perform source data verification and to monitor drug accountability. The visit intervals will depend on the outcome of the remote monitoring of the eCRFs, the trial site's recruitment rate and the compliance of the trial site to the protocol and GCP. The monitor must at least visit the sites every 8-12 weeks.

The monitor must be given direct access to source documents (original documents, data and records). Direct access includes permission to examine, analyse, verify and reproduce any record(s) and report(s) that are important to evaluation of the clinical trial. In addition the monitor should be available for discussions by telephone. Monitors must perform 100% source data verification of all data points.

Screening failure data must be completed.

The monitor will ensure that the eCRFs are completed.

15 Data management

Data management is the responsibility of Data Management, Novo Nordisk Head Quarters. Data management may be delegated under an agreement of transfer of responsibilities to another data management unit within Novo Nordisk or to an external contract research organisation.

The subject and the biological material obtained from the subject will be identified by subject number, trial site and trial identification number. Appropriate measures such as encryption or deletion will be enforced to protect the identity of human subjects in all presentations and publications as required by local/regional/national requirements.

Except for data from gene expression, proteomic profiling of serum/plasma markers, histopathology, and immunohistochemistry analyses, all data from biomarker assessments will be entered into the trial database at least in a processed form. Data from gene expression, proteomic analysis of serum/plasma markers, histopathology, and immunohistochemistry analyses will not be released at the time of trial database release.

Appropriate measures such as encryption of data files will be used to assure confidentiality of subject data when it is transmitted over open networks. In cases where electronic data capture is used, data stored on the Investigator's computer(s) and data during transfer will be secured by encryption.

Laboratory data will be transferred electronically from the central laboratory performing clinical analyses. The electronic laboratory data will be considered source data. In cases where laboratory data is transferred via non-secure electronic networks, data will be encrypted during transfer.

16 Evaluability of subjects for analysis

All subjects exposed to trial drug will be included in the safety analysis set used for analysis of safety data. All randomised and exposed subjects will be included in the intention to treat (ITT) analysis set used for the efficacy analysis. The per protocol (PP) analysis set will be comprised of subjects from the ITT analysis set who have fulfilled all inclusion and no exclusion criteria at entry, as well as no withdrawal criteria during trial. The PP analysis of the efficacy data will be viewed as a supportive analysis. The PK/PD analysis set used for the analysis of the PK/PD data will include subjects from the ITT analysis set who have not committed any protocol violations judged to interfere with the pharmacokinetics/pharmacodynamics of 109-0012.

The decision to exclude any subject or observation from statistical analysis is the joint responsibility of the International Trial Manager, Sponsor's Medical Expert and the Statistician. The subjects or observations to be excluded and the reason for their exclusion will be documented and signed by all relevant parties at a review of data prior to database release.

17 Statistical considerations

It is not acceptable that anyone, Investigator nor sponsor, makes any form of statistical comparisons between treatment groups before database release.

Novo Nordisk A/S will be responsible for the statistical analysis.

17.1 Sample size calculation

No formal power calculation has been performed in connection with the SD dose-escalation and MD dose-escalation phases. The number of subjects dosed within each cohort is based on safety. The objective is to expose the lowest possible number of subjects to the investigational drug, while still being able to assess safety, PK and PD data. Placebo will be used in order to distinguish between drug related effects and non-drug related effects.

The sample size calculation for the expansion cohort is based on an assumption of 60% of the Anti-IL-20 (109-0012) 100 mg/vial treated subjects and 10% of the placebo treated subjects, respectively, reaching a 75% reduction in PASI (PASI75), when comparing PASI, measured two weeks after last (fourth) dosing in the MD expansion phase, to baseline value measured prior to the first dose. Basing the comparison between the two treatment groups on a two sided Fisher's exact test, a 5 % significance level, and a 2:1 allocation ratio between Anti-IL-20 (109-0012) 100 mg/vial and placebo, including 26 subjects in the Anti-IL-20 (109-0012) 100 mg/vial group and 13 subjects in the placebo group should result in a power of 83%. Some additional sample size calculation using alternative assumptions are included in Table 17–1.

Table 17–1 Sample size calculation for the MD expansion phase

Active treatment	60% PASI75	55% PASI75	70% PASI75	70% PASI75	60% PASI75
Placebo	15% PASI75	10% PASI75	20% PASI75	10% PASI75	10% PASI75
	80%power with 32+16 subjects	80%power with 30+15 subjects	80%power with 28+14 subjects	90%power with 22+11 subjects	83%power with 26+13 subjects

17.2 Statistical methods

17.2.1 Primary endpoint

Primary endpoint for safety is adverse events and observed toxicity to Anti-IL-20 (109-0012) 100 mg/vial administration where the severity is assessed using NCI's CTCAE, version 3. All adverse events with onset after the first administration of trial drug will be included in the analysis.

The adverse events will be analysed using descriptive statistics. The adverse events will be summarised by trial phase (SD dose-escalation, MD dose-escalation or MD expansion phase), dose, MedDRA system organ class and MedDRA preferred term. The summary will additionally be presented by CTCAE grading (severity) and by relation. The descriptive statistics will include the number and percentage of subjects who experienced adverse events, the severity and the number of events. Adverse events will be listed by trial phase, dose and subject with information on severity according to the CTCAE criteria, relationship to trial drug and demographics.

17.2.2 Secondary endpoints

Efficacy:

- PASI (PASI75 is the primary endpoint for the MD expansion phase)
- PGA
- VAS (assessment of arthritis symptoms for subjects with psoriatic arthritis)
- QoL (MD expansion phase only)

PASI75, 75% reduction in PASI, will be assessed by comparing the measurement from the visit at trial Day 29 in the SD phase, and trial Day 57 in the MD phases, to baseline values measured prior to the first dose. PASI90 will be derived analogously.

PASI, PGA and VAS will be listed by trial phase, dose, and time. Additionally, PASI75 and PASI90 will be summarised by trial phase and dose, and PASI, PGA, VAS and QoL will be summarised by treatment group for the MD expansion phase.

Safety:

- Vital signs (blood pressure, pulse and temperature)
- ECG
- Laboratory safety parameters of haematology, biochemistry, hormones, lipids and urinalysis
- Immunogenicity (antibodies to 109-0012)
- Physical examination

The secondary safety endpoints will be analysed using descriptive statistics.

Pharmacokinetics:

The serum concentration-time data of 109-0012 will be analysed by non-compartmental analysis (NCA). If feasible, the following pharmacokinetic parameters will be derived from these data: Terminal half-life ($t_{½}$), maximal observed concentration (C_{max}), time to reach maximal concentration (t_{max}), accumulation index (R_{acc}) area under the curve in the dosing interval ($AUC_{0-\tau}$), area under the curve from time 0 to t (AUC_{0-t}) and area under the curve from time 0 to infinity (AUC). The terminal half-life will be calculated as $t_{½} = \ln(2)/\lambda_z$, where λ_z is the terminal rate constant. λ_z will be estimated as the slope from a linear regression with the natural logarithm to the concentration as the response variable, and time as the explanatory variable. Valid observations from the final part of the curve, which is approximately linear, will be used for the analysis.

Serum concentrations of 109-0012 will be summarised by trial phase and dose and listed by trial phase, dose and subject. The individual as well as the mean concentration-time profiles, by dose will be presented graphically. The pharmacokinetic parameters will be summarised by trial phase and dose and listed by trial phase, dose and subject.

Pharmacodynamics:

Pharmacodynamic biomarker assessments will be analysed using descriptive statistics.

17.3 Interim analysis

After each dose level, the bioanalysis results will be sent electronically (excel-file) for interim PK calculations to the representative in DMPK & Bioanalysis, Novo Nordisk A/S. The preliminary calculation of the PK parameters will be performed after each dose level and will be based on the nominal time points for the dose administration and PK sampling. The serum PK for 109-0012 will be calculated by NCA methods according to current local guidelines. The preliminary pharmacokinetic results will be available at the safety evaluation meetings and will support the dose-escalation decisions during the clinical phase of the trial. The dose escalation decisions will be based on safety and on PK data for all subjects in the current dose cohort through trial Day 15 in the SD dose-escalation phase, and through trial Day 57 in the MD dose-escalation phase. The decision to commence the MD dose-escalation phase will be taken after completion of the third dose level of the SD dose-escalation phase and will be based on safety and PK data for all available subjects. The PK data will be presented in such a manner that unblinding of the Safety Group is avoided.

After completion of the SD dose-escalation phase a partial data base release of the SD dose-escalation phase data will be done and the released data (safety, PK and efficacy) will be analysed using descriptive statistics. Furthermore, after the completion of the SD and MD dose-escalation

phases a decision, based on unblinded safety data will be taken, whether or not the trial should continue into the MD expansion phase. There will also be the possibility to go directly into a phase 2 dose-finding trial.

17.4 Explorative statistical analysis for pharmacogenetics and biomarkers

These analyses will be planned and performed after trial end when data has been unblinded.

17.5 PK and/or PD modelling

In addition to the NCA PK evaluation, an exploratory population PK/PD model will be developed. The details of the PK/PD modelling are described in the Modelling Analysis Plan (MAP), which will be prepared before FPFV. Model development will initially be based on interim data. Until data base release, the responsible biomodelling scientist will not take part in Study Safety Group meetings or disclose interim results to any other personnel. After data base release, final models will be created in which all relevant data decisions made at the release meeting have been implemented.

The below list of data will be explored for potential use in the PK/PD-model:

- Serum concentration of 109-0012 at all time points
- Serum concentration of IL-20 at all time points (free as well as amount bound by 109-0012)
- Calculated %neutralisation of serum IL-20 compared to predose IL-20
- Demographic covariates
    - Weight/BMI
    - Sex
- Sub-population: Psoriasis or psoriatic arthritis
- Biochemical assessments
- Pharmacogenomic biomarkers (when available)
- Skin histology
- Efficacy evaluations: PASI score etc. (section 17.2.2.)

For validation of the model and exclusion of outliers, please refer to the MAP. The results of the PK/PD model will be reported in a separate Biomodelling report.

18 Ethics

This is a first-in-man clinical trial with an antibody having a completely new molecular target, IL-20, a cytokine indicated to be involved in the development of psoriasis. The aim of the trial is to investigate the safety and tolerability of Anti-IL-20 (109-0012) 100 mg/vial after single doses, as well as after four repeated doses, in subjects with psoriasis.

The currently available treatments for moderate to severe psoriasis are all associated with more or less severe side effects. Furthermore many subjects experience loss of efficacy over time as well as rebound effects. Since there is an unmet medical need for safer, more effective and longer lasting treatment effects in subjects with moderate to severe chronic plaque psoriasis, it will be an incentive for subjects to participate in clinical trials which offer a potential benefit.

Pre-clinical safety studies in mice and monkeys have not revealed signs of toxicities with human relevance. Please refer to the IB, 1st Ed, and any updates hereof. However, there is a risk that adverse effects, related to Anti-IL-20 (109-0012) 100 mg/vial administration that have not been identified pre-clinically, can occur in humans. The theoretical adverse events that could potentially be related to administration of the trial product, include injection related events, and adverse events correlated with inhibiting the effect of IL-20, of which the importance to other immune responses are presently unknown.

A rational selection of the starting dose will be made. Thus, the starting dose selection will aim at achieving detectable, but not pharmacological active, concentrations of 109-0012. A cautious dose-escalation principle based on safety data through trial Week 5 will be applied in the SD, dose-escalation phase. In the MD dose-escalation phase a subsequent dose cohort will not be commenced until safety data from 2 weeks after the fourth and last dose have been evaluated, i.e. trial Week 9.

At the termination of the trial, the subject will consult with their Investigator to decide on the best available treatment.

The trial will be conducted in accordance with the Declaration of Helsinki.[35]

18.1 Informed consent form for trial subjects

In obtaining and documenting informed consent, the Investigator must comply with the applicable regulatory requirement(s) and adhere to the ICH GCP [34] and the requirements in the Declaration of Helsinki.[35]

Prior to any trial-related activity, the Investigator must give the subject oral and written information about the trial in a form that the subject can read and understand.

A voluntary, signed and personally dated, including time (if necessary), informed consent form will be obtained from the subject prior to any trial-related activity.

The written informed consent must be signed and personally dated, by the person who obtained the informed consent.

If information becomes available that may be relevant to the subject's willingness to continue participating in the trial, the Investigator must inform the subject in a timely manner, and a revised written informed consent must be obtained.

Samples for pharmacogenomic analyses will require a separate written information and informed consent will thus be provided to the subjects for this purpose. It will provide the possibility for the subject to abstain from genetic testing but still be able to participate in the non-genetic part of the trial.

18.2 Data handling

If the subject withdraws the previously given informed consent the subject's data will be handled as follows:

- Data collected will be used as part of the per protocol/intention to treat population.
- Safety events will be reported to International Product Safety, Novo Nordisk /regulatory authorities.

If data is used, it will always be in accordance with local law and subject to IRB/IEC approval.

18.3 Institutional review boards/independent ethics committee

Prior to commencement of the trial, the protocol, any amendments, subject information/informed consent form, any other written information to be provided to the subject, IB, information about payments and compensation available to subjects if not mentioned in the subject information, and other documents as required by the local IRB/IEC should be submitted. The submission letter should clearly identify which documents have been submitted to the IRB/IEC. Written approval/favourable opinion must be obtained from IRB/IEC prior to commencement of the trial.

During the trial, the Investigator must promptly in accordance with local requirements report the following to the IRB/IEC: Updates to IB, unexpected SAEs where a causal relationship cannot be ruled out, substantial amendments to the protocol, non-substantial amendments according to local requirements, deviations to the protocol implemented to eliminate immediate hazards to the subjects, new information that may affect adversely the safety of the subjects or the conduct of the

trial (including new risk/benefit analysis in case it will have an impact on the planned follow-up of the subjects), annually written summaries of the trial status and other documents as required by the local IRB/IEC.

Substantial amendments must not be implemented before approval/favourable opinion, unless necessary to eliminate hazards to the subjects.

The Investigator must maintain an accurate and complete record of all submissions made to the IRB/IEC. The records should be filed in the investigator's trial file and copies must be sent to Novo Nordisk.

18.4 Regulatory authorities

Regulatory authorities will receive the eIND, substantial/non-substantial amendments to the protocol, IND Safety Reports, and the clinical trial report according to national requirements.

19 Premature termination of the trial

The sponsor, Investigator or a pertinent regulatory authority may decide to stop the trial or part of the trial at any time, but agreement on procedures to be followed must be obtained.

If a trial is prematurely terminated or suspended, the Investigator should promptly inform the subjects and assure appropriate therapy and follow-up. Furthermore, the Investigator and/or sponsor should promptly inform the IEC/IRB and provide a detailed written explanation. The pertinent regulatory authorities should be informed according to national regulations.

If, after the termination of the trial, the risk/benefit analysis has changed, the new evaluation should be provided to the IEC/IRB in case it will have an impact on the planned follow-up of the subjects who have participated in the trial. If so, the actions needed to protect the subjects should be described.

20 Protocol compliance

Deviations from the protocol should not occur.

If deviations occur, the Investigator must inform the monitor and the implications of the deviation must be reviewed and discussed. Any deviation must be documented as required stating the reason and date, the action(s) taken, and the impact for the subject and/or the trial. The documentation must be kept in the investigator's trial file and the sponsor's trial master file.

21 Critical documents

Before the Investigator starts the trial (i.e. obtains informed consent from the first subject), the following documents must be available to Novo Nordisk:

- Regulatory approval and/or notification as required
- Curricula vitae of Investigator and Sub-investigator(s) (current, dated and signed and/or supported by an official regulatory document)
- Signed and dated agreement on the final protocol
- Signed and dated agreement on any substantial amendment(s), if applicable
- Approval/favourable opinion from IEC/IRB clearly identifying the documents reviewed: the protocol, any substantial amendments, subject information/informed consent form and any other written information to be provided to the subject, subject recruitment procedures
- Copy of IEC/IRB approved subject information/informed consent form/any other written information/advertisement
- List of IEC/IRB members/constitution
- Verification under disclosures per CFR of Financial Conflict of Interest
- FDA form 1572 for each Investigator
- FDA financial disclosure form or local equivalent as applicable
- Signed receipt of IB

22 Responsibilities

The Investigator is accountable for the conduct of the trial. If any tasks are delegated, the Investigator should maintain a list of appropriately qualified persons to whom he/she has delegated specified significant trial-related duties.

The medical care given to, and medical decisions made on behalf of, subjects should always be the responsibility of a qualified physician.

The Investigator will follow the instructions from Novo Nordisk when processing data.

The Investigator will take all necessary technical and organisational safety measures to prevent accidental or wrongful destruction, loss or deterioration of data. The Investigator will prevent any unauthorised access to data or any other processing of data against applicable law.

Upon request from Novo Nordisk, the Investigator will provide Novo Nordisk with the necessary information to enable Novo Nordisk to ensure that such technical and organisational safety measures have been taken.

It is the responsibility of the Principal Investigator to:

- Review/approve the Protocol
- Conduct the trial according to the approved Protocol, ICH-GCP and the Helsinki declaration
- Filing of Investigator Trial Master File (TMF)
- Review the final draft Clinical Trial Report

23 Reports and publications

The information obtained during the conduct of this trial is considered confidential and can be used by Novo Nordisk for regulatory purposes and for the general development of the trial product. All information supplied by Novo Nordisk in connection with this trial shall remain the sole property of Novo Nordisk and is to be considered confidential information. No confidential information shall be disclosed to others without prior written consent from Novo Nordisk. Such information shall not be used except in the performance of this trial. The information obtained during this trial may be made available to other physicians who are conducting other clinical trials with the trial product, if deemed necessary by Novo Nordisk.

The principal investigator, Alice Gottlieb, must review and sign the Clinical Trial Report.

23.1 Communication and publication

No permission to publish shall be granted to any clinical research organisation involved in the trial described in this protocol.

Novo Nordisk commits to communicating or otherwise making available for public disclosure results of trials regardless of outcome. Public disclosure includes publication of a paper in a scientific journal, abstract submission with a poster or oral presentation at a scientific meeting, or by other means.

Novo Nordisk reserves the right not to release data until specified milestones, e.g. a clinical trial report is available. This includes the right not to release interim results from clinical trials, because such results may lead to conclusions that are later shown to be incorrect.

At the end of the trial, one or more manuscripts for publication will be prepared in collaboration between Investigator(s) and Novo Nordisk. Novo Nordisk will not suppress or veto publications; however Novo Nordisk reserves the right to postpone publication and/or communication for a short time to protect intellectual property.

23.1.1 Authorship

Authorship of publications should be in accordance with guidelines from The International Committee of Medical Journal Editors’ Uniform Requirements (sometimes referred to as the Vancouver Criteria).[36]

23.1.2 Publication(s)

Within one year after first launch of the investigational trial product in any country (or one year after last subject last visit for trials finalised after obtaining marketing authorisation in any country) one of the following shall be posted at novotrials.com:

i) a summary of the results of the trial described in this protocol such as the clinical trial report synopsis

ii) the citation to the publication, or

iii) a stated intent to publish the results in a peer-reviewed journal. If the trial results have not been published within one year of the stated intent to publish, a summary of the results shall be posted to the database.

In all cases, the trial results shall be reported in an objective, accurate, balanced and complete manner, with a discussion of the strengths and limitations of the trial. All authors will be given the relevant statistical tables, figures, and reports needed to support the planned publication. In the event of any disagreement about the content of any publication, both the Investigators' and Novo Nordisk's opinions shall be fairly and sufficiently represented in the publication.

In a multi-centre trial based on the collaboration of all trial sites, any publication of results must acknowledge all trial sites.

Novo Nordisk maintains the right to be informed of any investigator plans for publication and to review any scientific paper, presentation, communication or other information concerning the investigation described in this protocol. Any such communication must be submitted in writing to the Novo Nordisk trial manager prior to submission for comments. Comments will be given within four weeks from receipt of the planned communication.

23.1.3 Site-specific publication(s) by Investigator(s)

For a multi-centre clinical trial, analyses based on single-site data usually have significant statistical limitations and frequently do not provide meaningful information for healthcare professionals or subjects, and therefore may not be supported by Novo Nordisk. It is Novo Nordisk's policy,that such individual reports do not precede the primary manuscript and should always reference the primary manuscript of the trial.

Novo Nordisk reserves the right to prior review of such publication and to ask for deferment of publication of individual site results until after the primary manuscript is accepted for publication.

23.2 Investigator access to data and review of results

As owners of the trial database, Novo Nordisk has discretion to determine who will have access to the database. Generally, trial databases are only made available to regulatory authorities.

24 Retention of clinical trial documentation

Subject notes must be kept for the maximum period permitted by the hospital, institution or private practice.

The Investigator must agree to archive the documentation pertaining to the trial in an archive after completion or discontinuation of the trial if not otherwise notified. The Investigator should not destroy any documents without prior permission from Novo Nordisk.

Clinical trial documentation must be retained until at least 2 years after the last approval of a marketing application in an ICH region and until there are no pending or contemplated marketing applications in an ICH region or at least 2 years have elapsed since the formal discontinuation of clinical development of the investigational product.

Novo Nordisk will maintain the documentation pertaining to the trial as long as the product is on the market and for a minimum of 20 years after the trial has been completed, or in accordance with national regulations if they require a longer retention period.

25 Indemnity statement

Novo Nordisk carries product liability for its products and liability assumed under the special laws, acts and/or guidelines for conducting clinical trials in any country, unless others have shown negligence.

Novo Nordisk assumes no liability in the event of negligence or any other liability by the clinics or doctors conducting experiments or by persons for whom the said clinic or doctors are responsible.

Novo Nordisk accepts liability in accordance with local laws, acts and guidances.

26 References

1. Luba KM, Stulberg DL. Chronic plaque psoriasis. Am Fam Physician 2006; 73(4):636-644.

2. Läkemedelsboken 2006. Apoteket AB, AO Konsult. Almqvist & Wiksell Tryckeri AB, Uppsala

3. Cassell S, Kavanaugh A. Psoriatic arthritis: pathogenesis and novel immunomodulatory approaches to treatment. J Immune Based Ther Vaccines 2005; 3:6.

4. Krueger G, Ellis CN. Psoriasis--recent advances in understanding its pathogenesis and treatment. J Am Acad Dermatol 2005; 53(1 Suppl 1):S94-100.

5. Lebwohl M. Psoriasis. Lancet 2003; 361(9364):1197-1204.

6. Mallbris L, Granath F, Hamsten A, Stahle M. Psoriasis is associated with lipid abnormalities at the onset of skin disease. J Am Acad Dermatol 2006; 54(4):614-621.

7. Duncan JI, Horrocks C, Ormerod AD et al. Soluble IL-2 receptor and CD25 cells in psoriasis: effects of cyclosporin A and PUVA therapy. Clin Exp Immunol 1991; 85(2):293-296.

8. van de Kerkhof PC, Wasel N, Kragballe K, Cambazard F, Murray S. A two-compound product containing calcipotriol and betamethasone dipropionate provides rapid, effective treatment of psoriasis vulgaris regardless of baseline disease severity. Dermatology 2005; 210(4):294-299.

9. Naldi L, Griffiths CE. Traditional therapies in the management of moderate to severe chronic plaque psoriasis: an assessment of the benefits and risks. Br J Dermatol 2005; 152(4):597-615.

10. Decision Resources; Psoriasis September 2006.

11. Hsing CH, Ho CL, Chang LY, Lee YL, Chuang SS, Chang MS. Tissue microarray analysis of interleukin-20 expression. Cytokine 2006; 35(1-2):44-52.

12. Wei CC, Chen WY, Wang YC et al. Detection of IL-20 and its receptors on psoriatic skin. Clin Immunol 2005; 117(1):65-72.

13. Romer J, Hasselager E, Norby PL, Steiniche T, Thorn CJ, Kragballe K. Epidermal overexpression of interleukin-19 and -20 mRNA in psoriatic skin disappears after short-term treatment with cyclosporine a or calcipotriol. J Invest Dermatol 2003; 121(6):1306-1311.

14. Blumberg H, Conklin D, Xu WF et al. Interleukin 20: discovery, receptor identification, and role in epidermal function. Cell 2001; 104(1):9-19.

15. Hunt DW, Boivin WA, Fairley LA et al. Ultraviolet B light stimulates interleukin-20 expression by human epithelial keratinocytes. Photochem Photobiol 2006; 82(5):1292-1300.

16. Otkjaer K, Kragballe K, Johansen C et al. IL-20 gene expression is induced by IL-1beta through mitogen-activated protein kinase and NF-kappaB-dependent mechanisms. J Invest Dermatol 2007; 127(6):1326-1336.

17. EMEA. Guideline on Clinical Investigations of Medicinal Products Indicated for the Treatment of Psoriasis. 2004.

18. EMEA. Guideline on Requirement for First-in-Man Clinical Trials for High Risk Medicinal Products. 2007.

19. Gibson R, Graham S, Lilienfeld A, Schuman L, Levin M, Swanson M. Epidemiology of diseases in adult males with leukemia. J Natl Cancer Inst 1976; 56(5):891-898.

20. Tavani A, La VC, Franceschi S, Serraino D, Carbone A. Medical history and risk of Hodgkin's and non-Hodgkin's lymphomas. Eur J Cancer Prev 2000; 9(1):59-64.

21. De AC, Drazen JM, Frizelle FA et al. Clinical trial registration: a statement from the International Committee of Medical Journal Editors. N Engl J Med 2004; 351(12):1250-1251.

22. Food and Drug Administration, Guidance for Industry, Information Program on Clinical Trials for Serious or Life-Threatening Diseases and Conditions, 2002. c

23. http://ctep.cancer.gov/forms/CTCAEv3.pdf. 2007.

24. Schmitt J, Wozel G. The psoriasis area and severity index is the adequate criterion to define severity in chronic plaque-type psoriasis. Dermatology 2005; 210(3):194-199.

25. Berth-Jones J, Grotzinger K, Rainville C et al. A study examining inter- and intrarater reliability of three scales for measuring severity of psoriasis: Psoriasis Area and Severity Index, Physician's Global Assessment and Lattice System Physician's Global Assessment. Br J Dermatol 2006; 155(4):707-713.

26. Feldman SR, Krueger GG. Psoriasis assessment tools in clinical trials. Ann Rheum Dis 2005; 64 Suppl 2:ii65-ii68.

27. Krueger GG, Langley RG, Finlay AY et al. Patient-reported outcomes of psoriasis improvement with etanercept therapy: results of a randomized phase III trial. Br J Dermatol 2005; 153(6):1192-1199.

28. Gottlieb AB, Chamian F, Masud S et al. TNF inhibition rapidly down-regulates multiple proinflammatory pathways in psoriasis plaques. J Immunol 2005; 175(4):2721-2729.

29. Lowes MA, Bowcock AM, Krueger JG. Pathogenesis and therapy of psoriasis. Nature 2007; 445(7130):866-873.

30. Leong TT, Fearon U, Veale DJ. Angiogenesis in psoriasis and psoriatic arthritis: clues to disease pathogenesis. Curr Rheumatol Rep 2005; 7(4):325-329.

31. Sa SM, Valdez PA, Wu J et al. The effects of IL-20 subfamily cytokines on reconstituted human epidermis suggest potential roles in cutaneous innate defense and pathogenic adaptive immunity in psoriasis. J Immunol 2007; 178(4):2229-2240.

32. Wang F, Lee E, Lowes MA et al. Prominent production of IL-20 by CD68+/CD11c+ myeloid-derived cells in psoriasis: Gene regulation and cellular effects. J Invest Dermatol 2006; 126(7):1590-1599.

33. Kingo K, Koks S, Nikopensius T, Silm H, Vasar E. Polymorphisms in the interleukin-20 gene: relationships to plaque-type psoriasis. Genes Immun 2004; 5(2):117-121.

34. International Conference on Harmonisation. ICH Harmonised Tripartite Guideline. Good Clinical Practice. 01-May-1996.

35. World Medical Association. Declaration of Helsinki. Ethical Principles for Medical Research Involving Human Subjects. 52nd WMA General Assembly, Edinburgh, Scotland, October 2000. Last amended with Note of Clarification on Paragraph 29 by the WMA General Assembly, Washington 2002, and Note of Clarification on Paragraph 30 by the WMA General assembly, Tokyo 2004.

36. Uniform requirements for manuscripts submitted to biomedical journals. International Committee of Medical Journal Editors. N Engl J Med 1997; 336(4):309-315.
